# Simulation of Cl^−^ Secretion in Epithelial Tissues: New Methodology Estimating Activity of Electro-Neutral Cl^−^ Transporter

**DOI:** 10.3389/fphys.2015.00370

**Published:** 2015-12-23

**Authors:** Kouhei Sasamoto, Naomi Niisato, Akiyuki Taruno, Yoshinori Marunaka

**Affiliations:** ^1^Department of Molecular Cell Physiology, Graduate School of Medical Science, Kyoto Prefectural University of MedicineKyoto, Japan; ^2^Department of Health and Sports Sciences, Faculty of Health and Medical Sciences, Kyoto Gakuen UniversityKameoka, Japan; ^3^Japan Institute for Food Education and Health, St. Agnes' UniversityKyoto, Japan; ^4^Department of Bio-Ionomics, Graduate School of Medical Science, Kyoto Prefectural University of MedicineKyoto, Japan

**Keywords:** Cl^−^ channel, Cl^−^ transporter, mathematical model, simulation, epithelial Cl^−^ secretion

## Abstract

Transcellular Cl^−^ secretion is, in general, mediated by two steps; (1) the entry step of Cl^−^ into the cytosolic space from the basolateral space across the basolateral membrane by Cl^−^ transporters, such as Na^+^-K^+^-2Cl^−^ cotransporter (NKCC1, an isoform of NKCC), and (2) the releasing step of Cl^−^ from the cytosolic space into the luminal (air) space across the apical membrane via Cl^−^ channels, such as cystic fibrosis transmembrane conductance regulator (CFTR) Cl^−^ channel. Transcellular Cl^−^ secretion has been characterized by using various experimental techniques. For example, measurements of short-circuit currents in the Ussing chamber and patch clamp techniques provide us information on transepithelial ion movements via transcellular pathway, transepithelial conductance, activity (open probability) of single channel, and whole cell currents. Although many investigators have tried to clarify roles of Cl^−^ channels and transporters located at the apical and basolateral membranes in transcellular Cl^−^ secretion, it is still unclear how Cl^−^ channels/transporters contribute to transcellular Cl^−^ secretion and are regulated by various stimuli such as Ca^2+^ and cAMP. In the present study, we simulate transcellular Cl^−^ secretion using mathematical models combined with electrophysiological measurements, providing information on contribution of Cl^−^ channels/transporters to transcellular Cl^−^ secretion, activity of electro-neutral ion transporters and how Cl^−^ channels/transporters are regulated.

## Introduction

Goblet cells located in airway surface epithelia and mucous cells of submucosal glands produce mucins, which are secreted into the airway space (Kim, 2012). Mucus layers formed by this secreted mucins covering airway epithelial surfaces trap pathogens such as bacteria and viruses in the mucus layers, which are removed by mucociliary clearance (Kim, 2012; Komatani-Tamiya et al., 2012). Transcellular Cl^−^ secretion in airway epithelial cells followed by paracellular Na^+^ transport (secretion) from the basolateral space to the apical space increases osmotic pressure in the apical space, resulting in water secretion (Asano et al., 2009; Kim et al., 2014; Marunaka, 2014a; Stanton et al., 2015). Diminution of water secretion driven by NaCl secretion elevates viscosity of mucins, causing dysfunction of mucociliary clearance (Kunzelmann and Schreiber, 2012). Thus, transcellular Cl^−^ secretion plays a crucial role in prevention from infection of bacteria and viruses, etc., by producing water secretion into the apical space, covering the apical surface of airway epithelial cells.

Transcellular Cl^−^ secretion in epithelial cells is generally mediated by two steps; (1) the entry step of Cl^−^ across the basolateral membrane by Cl^−^ transporters such as Na^+^-K^+^-2Cl^−^ cotransporter (NKCC1, an isoform of NKCC), and (2) the releasing step of Cl^−^ across the apical membrane via Cl^−^ channels such as the cystic fibrosis transmembrane conductance regulator (CFTR) Cl^−^ channel (Lee and Foskett, 2010; Li et al., 2012; Stölting et al., 2014; Sun et al., 2014a,b; Stanton et al., 2015). Transcellular Cl^−^ secretion across the epithelial tissue has been characterized by using the technique measuring short-circuit currents. Using blockers of Cl^−^ channels/transporters combined with the short-circuit current measurement technique, we can determine amounts of transepithelial Cl^−^ secretion (Marunaka, 2014a; Sun et al., 2014a,b). However, using this technique, we could not exactly determine how transcellular Cl^−^ secretion varies in magnitude and time due to modification of activity of Cl^−^ channels and transporters located at the apical and basolateral membranes. In the present study, we tried to clarify how transcellular Cl^−^ secretion varies in magnitude and time when the activity of Cl^−^ channels and transporters located at the apical and basolateral membranes changes using mathematical simulation with three parameters: (1) the entry step of Cl^−^ into the intracellular space from the basolateral space across the basolateral membrane by Cl^−^ transporters, (2) the releasing step of Cl^−^ from the intracellular space into the apical space across the apical membrane via Cl^−^ channels, and (3) the releasing step of Cl^−^ from the intracellular space to the basolateral space across the basolateral membrane via Cl^−^ channels (recycle, back flow/flux). This simulation method reported in the present study shows us how the activity of Cl^−^ channels and transporters located at the apical and basolateral membranes contributes to the transcellular Cl^−^ secretion, and the determination of the activity of electro-neutral Cl^−^ transporter such as NKCC1. Data have been partly reported in abstract form (Sasamoto et al., 2014).

## Methods

### Chemicals and materials

We obtained forskolin, daidzein, apigenin, genistein, protein kinase inhibitor 14–22 amide (PKI), NPPB (5-nitro-2-(3-phenylpropylamino)benzoic acid), nystatin, benzamil, and dimethyl sulfoxide (DMSO) from Sigma-Aldrich (St Louis, MO, USA), and epithelial A6 cells from American Type Culture Collection (ATCC). Forskolin (10 μM), daidzein (100 μM), apigenin (100 μM), genistein (100 μM), PKI (2 μM), NPPB (100 μM), nystatin (50 μM), and benzamil (10 μM) dissolved in DMSO were applied to the solution as the final concentration. The concentrations of forskolin, daidzein, apigenin, genistein, and PKI used in the present study were determined from the observations obtained in previous reports (Niisato et al., 1999), and these compounds were applied into both apical and basolateral solutions.

### Cell culture

A6 cells were derived from the kidney of *Xenopus laevis*, an amphibian, and cultured (passage 73–84) similar to our previous reports (Niisato and Marunaka, 1999; Sun et al., 2013, 2014a,b). To measure apical and basolateral Cl^−^ conductance (*G*_*A*_ and *G*_*B*_) and short-circuit current (*I*_*SC*_), we cultured A6 cells for 14 days on 6.5 mm Transwell-Clear permeable supports (0.33 cm^2^): *G*_*A*_ and *G*_*B*_ were actually measured in the area of 0.33 cm^2^. Volume of individual A6 cell was approximately 3.4 × 10^−15^ m^3^, and total volume of A6 cells cultured on Transwell-Clear permeable supports (0.33 cm^2^) was approximately 5.0 × 10^−10^ m^3^. The lateral membrane of A6 cells made tight junction expressing claudin-1, the width of which was less than 3 nm, showing the width of the paracellular space was less than 3 nm (Tokuda et al., 2008; Suzuki et al., 2009).

### Measurement of Cl^−^ conductance of apical and basolateral membranes (*G*_*A*_ and *G*_*B*_)

We transferred monolayers of A6 cells subcultured on tissue culture-treated Transwell filter cups to a modified Ussing chamber (Jim's Instrument, Iowa City, IA, USA) designed to hold the filter cup similar to another type of Ussing chamber (Marques et al., 2013), and continuously measured transepithelial potential difference (PD) by a high-impedance millivoltmeter (VCC-600, Physiologic Instrument, San Diego, CA, USA; Niisato and Marunaka, 1999; Sun et al., 2014b). We applied a pulse of +1 μA constant current every 10 s for 0.5 s to A6 monolayers under open-circuit conditions from the basolateral to the apical space, and calculated the conductance (G) from the ΔPD caused by the 1 μA constant-current pulse using Ohm's law (G = 1 μA/ΔPD mV: ΔPD had a positive value). We applied 100 μM NPPB (a non-selective Cl^−^ channel blocker, Niisato and Marunaka, 1999) to the apical or basolateral solution for detection of the NPPB-sensitive conductance of the apical or basolateral membrane used as the Cl^−^ conductance. We measured an NPPB-sensitive conductance (Niisato and Marunaka, 1999; Tokuda et al., 2007, 2008, 2009a,b, 2010). To detect the Cl^−^ conductance of the apical membrane, we measured the NPPB-sensitive conductance by applying 100 μM NPPB to the apical solution 45 min after addition of 50 μM nystatin to the basolateral membrane that permeabilized the basolateral membrane. To detect the Cl^−^ conductance of the basolateral membrane, we measured the NPPB-sensitive conductance by applying 100 μM NPPB to the basolateral solution 45 min after addition of 50 μM nystatin to the apical membrane that permeabilized the apical membrane (Niisato and Marunaka, 1999). We applied forskolin of 10 μM to both apical and basolateral spaces 30 min before addition of nystatin to detect the forskolin action on the membrane conductance. The values of measured PD were within the range of −1 to −30 mV depending on the experimental conditions.

### Measurement of short-circuit current (Isc)

We measured Isc in A6 cells according to the method as previously reported (Niisato and Marunaka, 1999; Sun et al., 2014b). To detect transepithelial Cl^−^ movements, we applied benzamil of 10 μM into the apical solution to block epithelial Na^+^ channel (ENaC) contributing to transepithelial Na^+^ absorption (Niisato and Marunaka, 1999). The value of *G*·*PD* (*I*_*SC*_) had a negative value, since PD had a negative value. However, most recently published articles present the Isc showing Cl^−^ secretion and also Na^+^ absorption as a positive current (Ikehara et al., 2014; Marunaka, 2014a,b). Therefore, in the present study, we present the Isc (Cl^−^ secretion) as a positive current.

### Solutions

The solution used in the present study contained (in mM) 120 NaCl, 3.5 KCl, 1 CaCl_2_, 1 MgCl_2_, 5 glucose, 10 HEPES with pH 7.4, since A6 cells used in the present study was derived from the kidney of an amphibian.

### Temperature

All experiments shown in the present study were performed at 24–25°C, which are physiological temperatures for amphibian, since A6 cells are amphibian cells.

### Data presentation

Values of *I*_*SC*_ and conductance are shown as the mean ± SEM. n means the number of experiments performed in the present study.

## Results

Many compounds show various time-dependent patterns in stimulation of transcellular Cl^−^ secretion in epithelial cells (Niisato et al., 1999; Hennig et al., 2008; Ao et al., 2013; Luo et al., 2013). Transcellular Cl^−^ secretion in epithelial cells is mediated by uptake and release of Cl^−^ into and from the intracellular space. To clarify the mechanism on uptake and release of Cl^−^ regulated by various types of compounds influencing transcellular Cl^−^ secretion, we propose a model of epithelial Cl^−^ secretion via the transcellular pathway by comparing this proposed model with experimental data on transcellular Cl^−^ secretion measured as *I*_*SC*_ in epithelial A6 cells.

### Model of transcellular Cl^−^ secretion in epithelial cells

**Figure 1:**
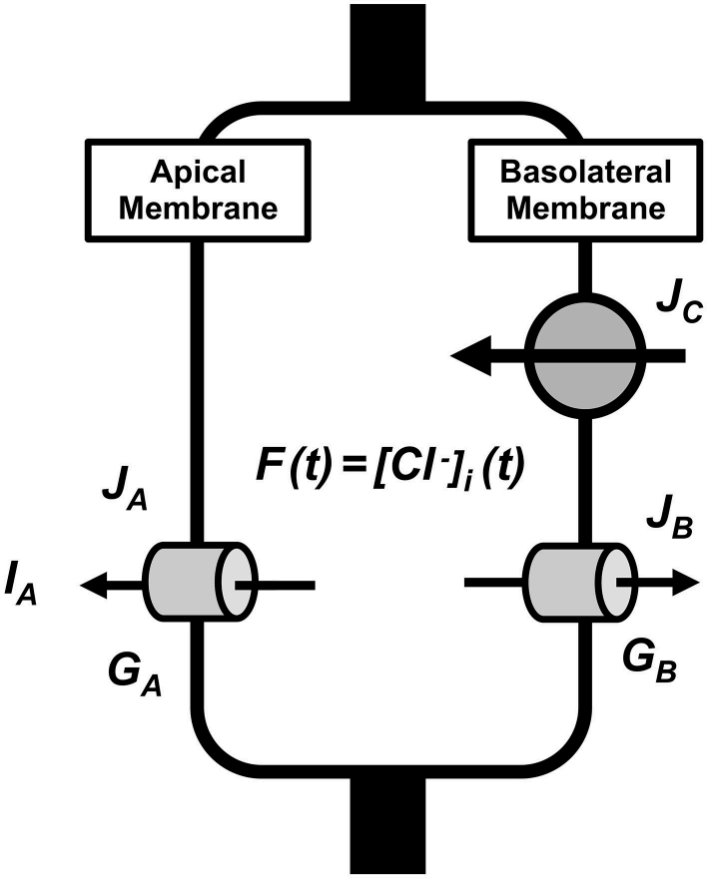
**A model of transcellular Cl^−^ secretion in epithelial tissues**. *F*(*t*) is the intracellular Cl^−^ concentration (${\left[Cl^{-}\right]}_{i}$) at time = *t*. *J*_*A*_ is the Cl^−^ flux through Cl^−^ releasing pathways (Cl^−^ channels) across the apical membrane, *J*_*B*_ is the Cl^−^ flux through Cl^−^ releasing pathways (Cl^−^ channels) across the basolateral membrane, and *J*_*C*_ is the Cl^−^ flux through Cl^−^ transporter uptaking Cl^−^ across the basolateral membrane. *G*_*A*_ and *G*_*B*_ are respectively the apical and basolateral Cl^−^ conductance.

The parameters used in the present study are listed in Table 1. Figure 1 describes a model of transcellular Cl^−^ secretion in epithelial tissues. This model contains three Cl^−^ moving pathways between the intracellular and extracellular spaces across the apical and basolateral membranes: **(1)** a Cl^−^ releasing pathway from the intracellular space into the apical space, such as Cl^−^ channels, across the apical membrane (**Pathway A** contributing to Cl^−^ secretion as a passive Cl^−^ moving pathway driven by electrochemical potential of Cl^−^ between the intracellular and apical spaces across the apical membrane); **(2)** a Cl^−^ releasing pathway from the intracellular space into the basolateral space, such as Cl^−^ channels, across the basolateral membrane (**Pathway B** not contributing to Cl^−^ secretion as a passive Cl^−^ moving pathway driven by electrochemical potential of Cl^−^ between the intracellular and basolateral spaces across the basolateral membrane); **(3)** a Cl^−^ uptake pathway from the basolateral space into the intracellular space, such as NKCC1, across the basolateral membrane (**Pathway C** partially, but not all, contributing to Cl^−^ secretion as an active Cl^−^ moving pathway, such as NKCC1, driven by electrochemical potential of Na^+^ between the intracellular and basolateral spaces across the basolateral membrane). The transcellular Cl^−^ secretion consists of the following pathways: **(1)** Cl^−^ is first taken up into the intracellular space via **Pathway C**; **(2)** Cl^−^ taken up into the intracellular space by **Pathway C** is respectively released into the apical and basolateral spaces via **Pathways A and B**; Cl^−^ taken up by **Pathway C** released into the apical space via **Pathway A** only contributes to the transcellular Cl^−^ secretion.

**Table 1:** **Definition of characters**.

**Character**	**Definition**
α	Rate constant converting from the inactive form (*CT*_*Inact*_) to the active form (*CT*_*Act*_)
β	Rate constant converting from the active form (*CT*_*Act*_) to the inactive form (*CT*_*Inact*_)
${\left[{\text{Cl}}^{-}\right]}_{\text{i}}$	Intracellular Cl^−^ concentration
${\left[{\text{Cl}}^{-}\right]}_{\text{o}}$	Extracellular Cl^−^ concentration
*CT*	Cl^−^ transporters
*CT*_*Act*_	Active form of Cl^−^ transporters
*CT*_*Inact*_	Inactive form of Cl^−^ transporters
*CT*_*Act*_(*t*)	Amount of active form of Cl^−^ transporters (*CT*_*Act*_) at time of *t*
*CT*_*Inact*_(*t*)	Amount of inactive form of Cl^−^ transporters (*CT*_*Inact*_) at time of *t*
*CT*_*T*_	Total amount of Cl^−^ transporters
Cv	Cell volume
F	Faraday constant
*f*(*t*)	Intracellular Cl^−^ concentration at time = *t*
*G*_*A*_	Apical Cl^−^ conductance
*G*_*B*_	Basolateral Cl^−^ conductance
*I*_*A*_(*t*)	Cl^−^ current (secretion) across apical membrane at time = *t*
*I*_*SC*_	Short-circuit current
j	Amount of flux carried by Cl^−^ transporters (*CT*_*Act*_)/second
*J*_*A*_	Cl^−^ efflux via Cl^−^ channels located at apical membrane/unit apical membrane area
*J*_*B*_	Cl^−^ efflux via Cl^−^ channels located at basolateral membrane/unit basolateral membrane area
*J*_*C*_	Cl^−^ transport via Cl^−^ transporters (*CT*) located at basolateral membrane/unit basolateral membrane area
*P*_*A*_	Apical Cl^−^ permeability/unit apical membrane area
*P*_*B*_	Basolateral Cl^−^ permeability/unit apical membrane area
*PD*	Potential difference
R	Gas constant
S_A_	Area of apical membrane
S_B_	Area of basolateral membrane
T	Absolute temperature
*V*	Membrane potential
Z_Cl_	Charge contained in Cl^−^

Each character with superscripted 0 means its value under the basal condition. Each character with superscripted ∞ means its value at a steady state after application of compounds affecting transcellular Cl^−^ secretion.

We can, in general, estimate the transcellular Cl^−^ secretion measuring short-circuit currents in Ussing chamber (Ussing and Zerahn, 1951). Therefore, we consider a model of transcellular Cl^−^ secretion measured under a short-circuit (voltage clamp) condition with the apical membrane potential identical to the basolateral membrane potential and all ionic compositions of the apical solution identical to those of basolateral solution.

Based on characteristics of Cl^−^ movements described in a model (Figure 1), we defined Cl^−^ effluxes as positive values. To clearly show results, we describe how to obtain equations applied in the present study in Appendix: the equation's number shown in text compatible with that in Appendix. We define the intracellular Cl^−^ concentration (${\left[{Cl}^{-}\right]}_{i}$) at time = *t* as *f*(*t*) [see Equation (A13) in Appendix], where *t* is time after application of compounds affecting the Cl^−^ movements across the apical and/or basolateral membranes of epithelial cells. We show *I*_*SC*_ (transepithelial Cl^−^ secretion) at time = *t* as *I*_*A*_(*t*) [see Equation (A18) in Appendix]. Table 1 shows meanings of parameters used in the present study.

#### A case where Cl^−^ uptake via a pathway (*J*_*C*_) is constant $\left(J_{C}=J_{C}^{0}:\;CT_{Act}=CT_{Act}^{0}\right)$ and the membrane potential (*V*) is not changed (*V* = *V*^0^) after application of compounds affecting intracellular environments modifying apical and/or basolateral Cl^−^ conductance

These calculations indicate that: (1) an instantaneous Cl^−^ secretion (*I*_*A*_(0)) just after application of compounds affecting just *G*_*A*_ and/or *G*_*B*_ depends on a change in *G*_*A*_ but not *G*_*B*_; (2) a Cl^−^ secretion at a steady state, *I*_*A*_(∞), depends upon both changes in *G*_*A*_ and *G*_*B*_; (3) a transition Cl^−^ secretion occurs in response to changes in *G*_*A*_ and *G*_*B*_ time dependently with a time constant (τ) shown in Equation (A14). We further show some concrete examples of Cl^−^ secretion responses to changes in *G*_*A*_ and/or *G*_*B*_.

#### A case under a condition with a relative change in an apical Cl^−^ conductance larger than that in the basolateral Cl^−^ conductance $\left(\frac{G_{A}}{G_{A}^{0}}>\frac{G_{B}}{G_{B}^{0}}\right)$ without any change in Cl^−^ uptake across the basolateral membrane $\left(J_{C}=J_{C}^{0}:CT_{Act}=CT_{Act}^{0}\right)$ or membrane potential (*V* = *V*^0^)

**Figure 2:**
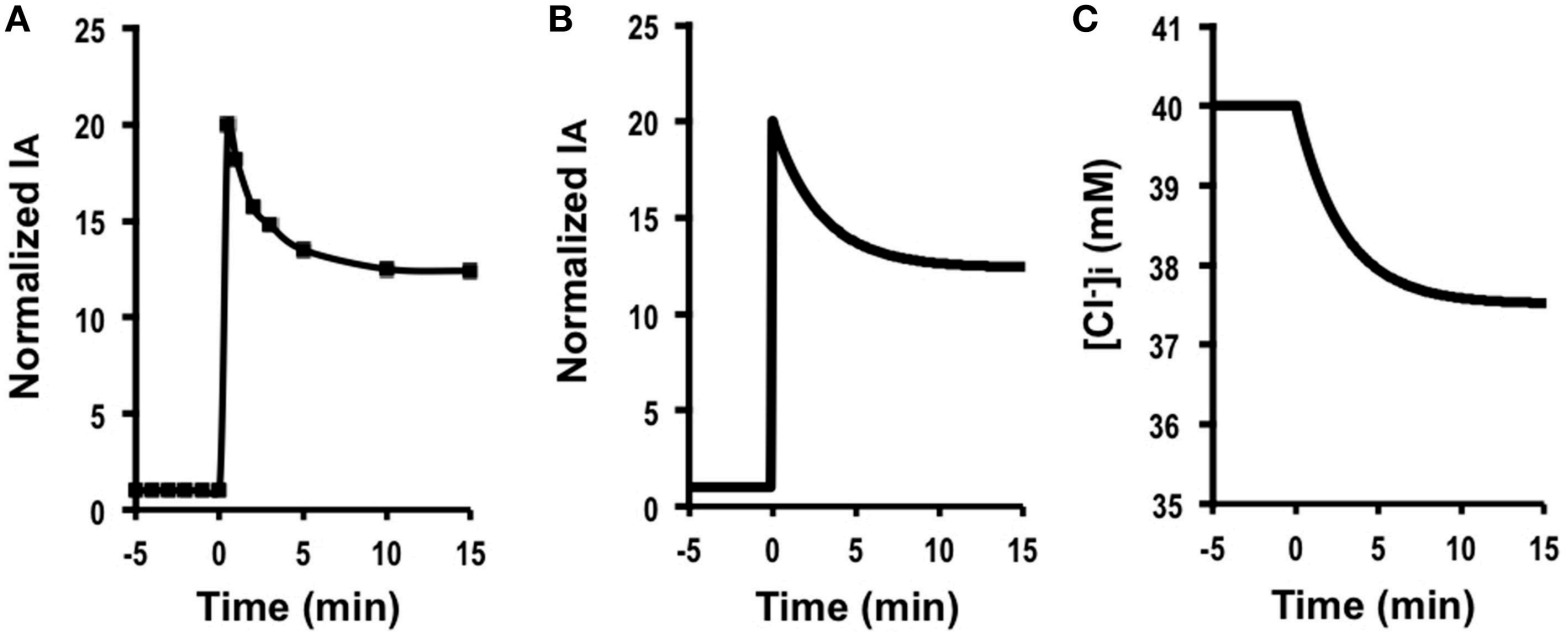
**The effect of forskolin in the presence of PKI on Cl^−^ secretion (*I*_*A*_) and simulation of Cl^−^ secretion (*I*_*A*_) and ${\left[Cl^{-}\right]}_{i}$ under a condition with only a change in the apical Cl^−^ conductance ($G_{A}=20\;G_{A}^{0}$) without any change in the basolateral Cl^−^ conductance ($G_{B}=G_{B}^{0}$), Cl^−^ uptake across the basolateral membrane ($J_{C}=J_{C}^{0}$) or membrane potential (*V* = *V*^0^) based on $G_{B}^{0}=30\;G_{A}^{0}$: $G_{A}=20\;G_{A}^{0}$, $G_{B}=G_{B}^{0}$, $G_{B}^{0}=30\;G_{A}^{0}$, $\frac{G_{A}}{{G^{0}}_{A}}>\frac{G_{B}}{G_{B}^{0}},J_{C}=J_{C}^{0},V=V^{0}$**. **(A)** The effect of forskolin in the presence of PKI on Cl^−^ secretion (*I*_*A*_). Forskolin (10 μM) applied to the apical and basolateral solutions (at time = 0 min in **A**) 10 min after addition of 2 μM PKI to the apical and basolateral solutions induced an increase in *I*_*A*_ followed by a decrease reaching a steady level larger than the basal one. **(B)** Simulation of *I*_*A*_ shown in **(A)**. A stimulant is applied at time = 0 min. $I_{A}\left(0\right)=20\;I_{A}^{0}$ [see Equation (A18)]. Then, Cl^−^ secretion gradually decreases, reaching a steady state; $I_{A}=12.4\;I_{A}^{0}$ [see Equation (A18)]. **(C)** Simulation of ${\left[Cl^{-}\right]}_{i}$ in cells secreting Cl^−^ secretion (*I*_*A*_) shown in **(B)**. A stimulant is applied at time = 0 min. ${\left[Cl^{-}\right]}_{i}$ gradually decreases.

*A case under a condition with only a change in the apical Cl^−^ conductance* $\left({\text{G}}_{\text{A}}=20{\text{G}}_{\text{A}}^{\text{0}}\right):{\text{G}}_{\text{A}}=20{\text{G}}_{\text{A}}^{\text{0}},\;{\text{G}}_{\text{B}}={\text{G}}_{\text{B}}^{\text{0}},\;{\text{G}}_{\text{B}}^{\text{0}}=\text{30}{\text{G}}_{\text{A}}^{\text{0}},\;\frac{{\text{G}}_{\text{A}}}{{\text{G}}_{\text{A}}^{\text{0}}}>\frac{{\text{G}}_{\text{B}}}{{\text{G}}_{\text{B}}^{\text{0}}},\;{\text{J}}_{\text{C}}={\text{J}}_{\text{C}}^{\text{0}}\;\left({\text{CT}}_{\text{Act}}={\text{CT}}_{\text{Act}}^{\text{0}}\right),\;\text{V}=\;{\text{V}}^{\text{0}}$. Figure 2A shows *I*_*A*_ (Cl^−^ secretion) in A6 cells treated with 10 μM forskolin in the presence of 2 μM protein kinase inhibitor 14–22 amide (PKI). Forskolin induced a transient increase in *I*_*A*_ followed by a declining phase, reaching a steady level (Figure 2A). Figure 2B shows simulation of *I*_*A*_ shown in Figure 2A: *I*_*A*_ increases in response to a 20-fold increase in apical Cl^−^ conductance ($G_{A}=20\;G_{A}^{0}$) without any change in basolateral Cl^−^ conductance ($G_{B}=\;G_{B}^{0}$). The instantaneous Cl^−^ secretion (*I*_*A*_ (0)) just after a change in *G*_*A*_ without any change in *G*_*B*_ is 20-fold larger than $I_{A}^{0}$ [Equation (A18)]. Then, Cl^−^ secretion gradually decreases, reaching a steady state; *I*_*A*_ (∞) is 12.4-fold larger than $I_{A}^{0}$ (see Equation A18). If we expect to induce continuous stimulation of Cl^−^ secretion, we should apply any compounds providing with larger activation on apical Cl^−^ channels than on basolateral Cl^−^ channels. τ of Cl^−^ secretion transition in response to a change in apical Cl^−^ conductance ($G_{B}^{0}=30\;G_{A}^{0},\;G_{A}=20\;G_{A}^{0},\;G_{B}=\;G_{B}^{0}$) obtained from Equation (A14) is $\frac{1}{50}\frac{\text{CvF}\left(\exp\left(\frac{FV^{0}}{RT}\right)-1\right){\left[{\text{Cl}}^{-}\right]}_{o}}{V^{0}G_{A}^{0}}$.

Simulated Cl^−^ secretion under this condition shows a transient increase followed by a decline toward to a steady state (Figure 2B). The simulated Cl^−^ secretion pattern shown in Figure 2B was similarly observed in carbachol-stimulated Cl^−^ secretion (Hendrick et al., 2014). This means that even if carbachol would activate the Cl^−^ channel located at the basolateral membrane, the action of carbachol on the basolateral Cl^−^ channel would be smaller than that on the apical Cl^−^ channel $\frac{G_{A}}{G_{A}^{0}}>\frac{G_{B}}{G_{B}^{0}}$. Combining the simulation and the experimental observation shown in a previous report (Hendrick et al., 2014), we could obtain more information on modulation of the Cl^−^ channels/transporters compared with only the experimental observation. Figure 2C shows a simulated change in [*Cl*${}^{-}{]}_{i}$ of the cells secreting Cl^−^ presented in Figure 2B. This simulation indicates that [*Cl*${}^{-}{]}_{i}$ decreases with a time course (Figure 2C) same as that in the declining phase in Cl^−^ secretion following the transient increase (Figure 2B), although [*Cl*${}^{-}{]}_{i}$ shows no transient change unlike Cl^−^ secretion (*I*_*A*_; see Figures 2B,C).

#### A case where both the apical Cl^−^ conductance $\left(G_{A}=2.5G_{A}^{0}\right)$ and the basolateral Cl^−^ conductance $\left(G_{B}=1.5G_{B}^{0}\right)$ increase, but a relative change in an apical Cl^−^ conductance is larger than that in the basolateral Cl^−^ $\left(\frac{G_{A}}{G_{A}^{0}}>\frac{G_{B}}{G_{B}^{0}}\right)$: $G_{A}=2.5\;G_{A}^{0}$, $G_{B}=1.5\;G_{B}^{0},\;G_{B}^{0}=30G_{A}^{0}$, $\frac{G_{A}}{G_{A}^{0}}>\frac{G_{B}}{G_{B}^{0}}$, $J_{C}=\;J_{C}^{0}\;\left(CT_{Act}=CT_{Act}^{0}\right)$, *V* = *V*^0^.

For example, if a condition, $G_{A}=2.5\;G_{A}^{0},\;G_{B}=1.5\;G_{B}^{0}$ and $G_{B}^{0}=30G_{A}^{0}$ (a relative change in *G*_*A*_ is larger than that in *G*_*B*_), is considered, the instantaneous Cl^−^ secretion (*I*_*A*_ (0)) is 2.5-fold larger than $I_{A}^{0}$ [see Equation (A18)]. Then, Cl^−^ secretion gradually decreases, reaching a steady state; *I*_*A*_(∞) is $\frac{77.5}{47.5}$ -fold (about 1.63-fold) larger than $I_{A}^{0}$ (see Equation A18). τ of Cl^−^ secretion transition in response to a change in apical Cl^−^ conductance ($G_{A}=2.5\;G_{A}^{0},G_{B}=1.5\;G_{B}^{0}$, and $G_{B}^{0}=30\;G_{A}^{0}$) obtained from Equation (A14) is $\frac{1}{47.5}\frac{\text{CvF}\left(\exp\left(\frac{\text{F}V^{0}}{\text{RT}}\right)-1\right){\left[{\text{Cl}}^{-}\right]}_{\text{o}}}{V^{0}G_{A}^{0}}$. Hollenhorst et al. have reported an observation on acetylcholine-stimulated Cl^−^ secretion (Hollenhorst et al., 2012) similar to this simulation.

In this case, $\frac{G_{A}}{G_{A}^{0}}>\frac{G_{B}}{G_{B}^{0}}$ even without any changes in $J_{C}\;\left(J_{C}=J_{C}^{0}:CT_{Act}=CT_{Act}^{0}\right)$, *I*_*A*_(∞) is larger than $I_{A}^{0}$. This case indicates a time course of transcellular Cl^−^ secretion similar to that shown in Figure 2B. Although we could not determine if the basolateral Cl^−^ conductance changes, this simulation leads us to conclude that the stimulatory action of acetylcholine on Cl^−^ secretion (Hollenhorst et al., 2012) is mediated through activation of the apical Cl^−^ channel that is larger than that of the basolateral Cl^−^ channel $\left(\frac{G_{A}}{G_{A}^{0}}\;>\;\frac{G_{B}}{G_{B}^{0}}\right)$.

#### A case under a condition with the same extent increases in both the apical Cl^−^ conductance and the basolateral conductance $\left(\frac{G_{A}}{G_{A}^{0}}=\frac{G_{B}}{G_{B}^{0}}\right)$ without any change in Cl^−^ uptake across the basolateral membrane $\left(J_{C}=J_{C}^{0}:CT_{Act}=CT_{Act}^{0}\right)$ or membrane potential (*V* = *V*^0^) based on $G_{B}^{0}=30\;G_{A}^{0}$; e.g., $G_{A}=18\;G_{A}^{0}$, $G_{B}=18\;G_{B}^{0},G_{B}^{0}=30\;G_{A}^{0}$, $\frac{G_{A}}{G_{A}^{0}}=\frac{G_{B}}{G_{B}^{0}}$, $J_{C}=J_{C}^{0}\left({CT}_{Act}={CT}_{Act}^{0}\right)$, *V* = *V*^0^

**Figure 3:**
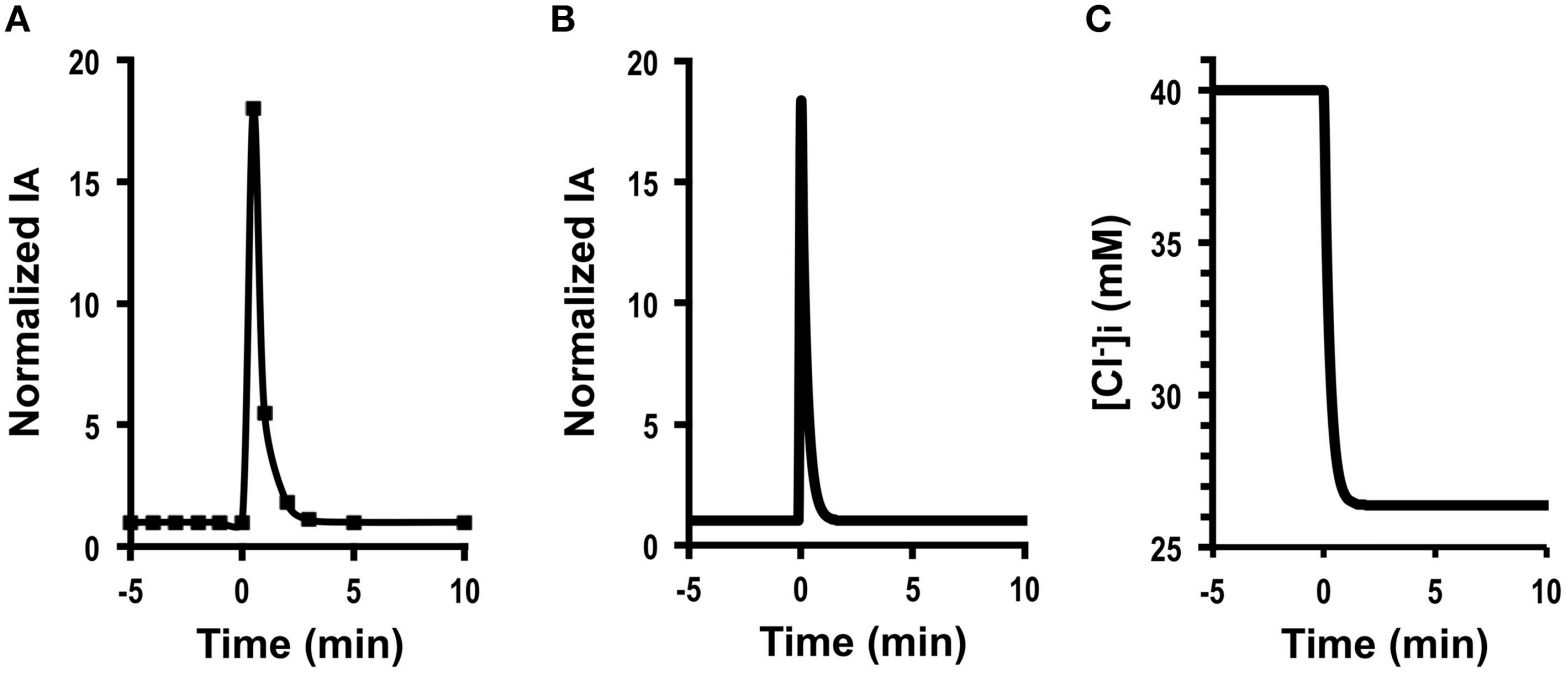
**The effect of daidzein on Cl^−^ secretion (*I*_*A*_) and simulation of Cl^−^ secretion (*I*_*A*_) and ${\left[{Cl}^{-}\right]}_{i}$ under a condition with the same extent increases in both the apical Cl^−^ and basolateral Cl^−^ conductance $\left(\frac{G_{A}}{G_{A}^{0}}=\frac{G_{B}}{G_{B}^{0}}\right)$ without any change in Cl^−^ uptake across the basolateral membrane ($J_{C}=J_{C}^{0}$) or membrane potential (*V* = *V*^0^): e.g., $G_{A}=18\;G_{A}^{0}$, $G_{B}=18\;G_{B}^{0}$, $G_{B}^{0}=30\;G_{A}^{0}$, $\frac{G_{A}}{G_{A}^{0}}=\frac{G_{B}}{G_{B}^{0}}$, $J_{C}=J_{C}^{0}$, *V* = *V*^0^**. **(A)** The effect of daidzein on Cl^−^ secretion (*I*_*A*_). Daidzein (100 μM) applied to the apical and basolateral solutions at time = 0 min induced an increase in *I*_*A*_ followed by a decrease reaching a steady level identical to the basal one. **(B)** Simulation of *I*_*A*_ shown in **(A)**. A stimulant is applied at time = 0 min. $I_{A}\;\left(0\right)=18\;I_{A}^{0}$ [see Equation (A18)]. Then, Cl^−^ secretion gradually decreases, reaching a steady state; $I_{A}\;\left(\infty \right)=I_{A}^{0}$ [see Equation (A18)]. **(C)** Simulation of [*Cl*${}^{-}{]}_{i}$ in cells secreting Cl^−^ secretion (*I*_*A*_) shown in **(B)**. A stimulant is applied at time = 0 min. [*Cl*${}^{-}{]}_{i}$ decreases with time after application of stimulant.

Figure 3A shows Cl^−^ secretion in A6 cells treated with 100 μM daidzein: daidzein induced a transient increase in *I*_*A*_ followed by a decrease, reaching a steady state identical to the basal one. Figure 3B shows simulation of *I*_*A*_ in daidzein-treated A6 cells: *I*_*A*_ increases in response to 18-fold elevation in apical and basolateral Cl^−^ conductance ($G_{A}=18\;G_{A}^{0},\;G_{B}=18\;G_{B}^{0}$; the same extent increases in relative changes in *G*_*A*_ and *G*_*B*_). The instantaneous Cl^−^ secretion (*I*_*A*_ (0)) just after increases in *G*_*A*_ and *G*_*B*_ is 18-fold larger than $I_{A}^{0}$ [see Equation (A18)]. Then, Cl^−^ secretion gradually decreases, reaching a steady state; *I*_*A*_ (∞) is identical to an initial value of Cl^−^ secretion [$I_{A}^{0}$; $I_{A}\;\left(\infty \right)=I_{A}^{0}$; see Equation (A18)]. τ of Cl^−^ secretion transition in response to a change in apical and basolateral Cl^−^ conductance ($G_{A}=18\;G_{A}^{0}$, $G_{B}=\;18\;G_{B}^{0}$, and $G_{B}^{0}=30\;G_{A}^{0}$) obtained from Equation (A14) is $\frac{1}{558}\;\frac{\text{CvF}\left(\exp\left(\frac{\text{F}V^{0}}{\text{RT}}\right)-1\right){\left[{\text{Cl}}^{-}\right]}_{\text{o}}}{V^{0}G_{A}^{0}}$.

These results indicate an interesting phenomenon that stimulants increasing apical and basolateral Cl^−^ conductance to the same extent ($G_{A}=18\;G_{A}^{0}$, $G_{B}=\;18\;G_{B}^{0}$) have no influence on a steady-state Cl^−^ secretion (*I*_*A*_ (∞) = *I*_*A*_ (0)) but induces only transient stimulation to Cl^−^ secretion (an 18-fold increase). Figure 3C shows a simulated change in ${\left[{Cl}^{-}\right]}_{i}$ of the cells secreting Cl^−^ presented in Figure 3B. The time course of ${\left[{Cl}^{-}\right]}_{i}$ decrease is same as that at the declining phase of Cl^−^ secretion following a transient increase, although ${\left[{Cl}^{-}\right]}_{i}$ shows no instantaneous change just after a change in *G*_*A*_ (see Figures 3B,C). A similar phenomenon is observed in Cl^−^ secretion in cells treated with formaldehyde, bile acids, or *Pasteurella multocida* toxin (Hennig et al., 2008; Ao et al., 2013; Luo et al., 2013), suggesting that formaldehyde, bile acids, or *Pasteurella multocida* toxin would activate the apical and basolateral Cl^−^ channels in the same level $\left(\frac{G_{A}}{G_{A}^{0}}=\frac{G_{B}}{G_{B}^{0}}\right)$.

#### A case under a condition with a relative change in the apical Cl^−^ conductance smaller than that in the basolateral Cl^−^ $\left(\frac{G_{A}}{G_{A}^{0}}<\frac{G_{B}}{G_{B}^{0}}\right)$ without any change in Cl^−^ uptake across the basolateral membrane $\left(J_{C}=J_{C}^{0}:\;{CT}_{Act}={CT}_{Act}^{0}\right)$ or membrane potential (*V* = *V*^0^): e.g., $G_{A}=G_{A}^{0},G_{B}=2\;G_{B}^{0},G_{B}^{0}=30\;G_{A}^{0},\frac{G_{A}}{G_{A}^{0}}<\frac{G_{B}}{G_{B}^{0}},J_{C}=J_{C}^{0}\left({CT}_{Act}={CT}_{Act}^{0}\right),V=V^{0}$

**Figure 4:**
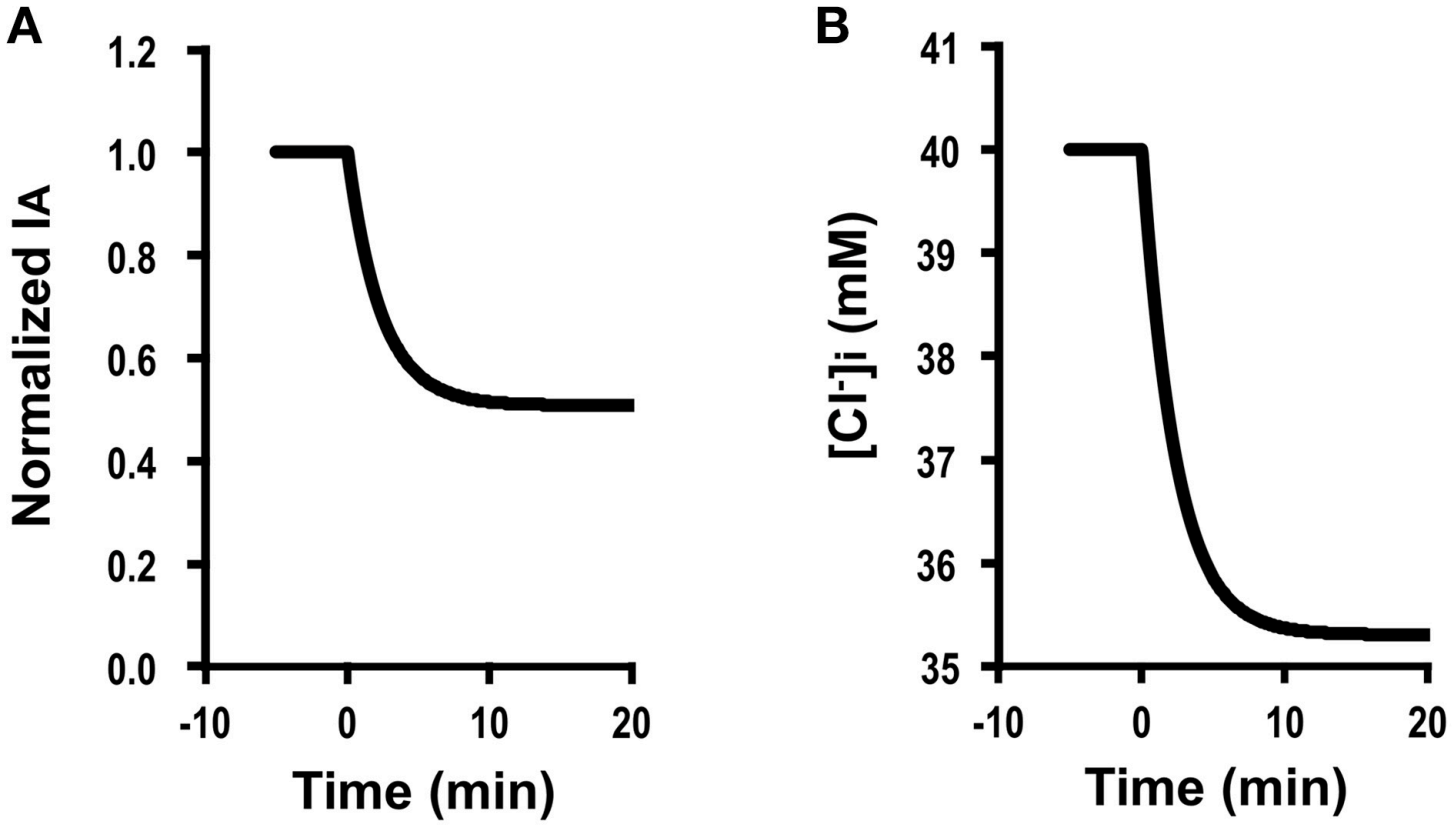
**Simulation of Cl^−^ secretion (*I*_*A*_) and ${\left[{Cl}^{-}\right]}_{i}$ under a condition with a relative change in the apical Cl^−^ conductance smaller than that in the basolateral Cl^−^$\left(\frac{G_{A}}{G_{A}^{0}}<\frac{G_{B}}{G_{B}^{0}}\right)$ without Cl^−^ uptake across the basolateral membrane ($J_{C}=J_{C}^{0}$) or membrane potential (*V* = *V*^0^): e.g., $G_{A}=G_{A}^{0}$, $G_{B}=2\;G_{B}^{0}$, $G_{B}^{0}=30\;G_{A}^{0}$, $\frac{G_{A}}{G_{A}^{0}}<\frac{G_{B}}{G_{B}^{0}}$, $J_{C}=J_{C}^{0}$, *V* = *V*^0^**. **(A)** Simulation of *I*_*A*_. A stimulant is applied at time =0 min. $I_{A}\;\left(0\right)=I_{A}^{0}$ [see Equation (A18)]. Then, Cl^−^ secretion gradually decreases reaching a steady state; $I_{A}\left(\infty \right)=\frac{31}{61}I_{A}^{0}$ [about 0.5-fold of $I_{A}^{0}$; see Equation (A18)]. **(B)** Simulation of ${\left[Cl^{-}\right]}_{i}$ in cells secreting Cl^−^ secretion (*I*_*A*_) shown in **(A)**. A stimulant is applied at time = 0 min. ${\left[Cl^{-}\right]}_{i}$ gradually decreases.

Figure 4A shows the time course of Cl^−^ secretion in response to two-fold increases in basolateral Cl^−^ conductance with no change in apical Cl^−^ conductance ($G_{A}=G_{A}^{0}$, $G_{B}=\;2\;G_{B}^{0}$). The instantaneous Cl^−^ secretion (*I*_*A*_(0)) just after an increase in *G*_*B*_ without any change in *G*_*A*_ does not change but remains at an identical level to $I_{A}^{0}$ (see Equation A18). Then, Cl^−^ secretion gradually decreases reaching a steady state; *I*_*A*_(∞) is smaller than an initial value of Cl^−^ secretion ($I_{A}^{0}$) [$I_{A}\left(\infty \right)=\frac{31}{61}I_{A}^{0}$ (about 0.5-fold of $I_{A}^{0}$); see Equation (A18)] in response to an increase in *G*_*B*_ with no change in *G*_*A*_. τ of Cl^−^ secretion transition in response to a change in basolateral Cl^−^ conductance ($G_{A}=G_{A}^{0}$, $G_{B}=\;{2\;G}_{B}^{0}$, and $G_{B}^{0}=30\;G_{A}^{0}$) obtained from Equation (A14) is $\frac{1}{61}\frac{\text{CvF}\left(\exp\left(\frac{\text{F}V^{0}}{\text{RT}}\right)-1\right){\left[{\text{Cl}}^{-}\right]}_{\text{o}}}{V^{0}G_{A}^{0}}$.

Figure 4B shows a simulated change in ${\left[{Cl}^{-}\right]}_{i}$ of the cells secreting Cl^−^ presented in Figure 4A. The time course of ${\left[{Cl}^{-}\right]}_{i}$ decrease (Figure 4B) is same as that of the declining phase of Cl^−^ secretion (Figure 4A). These results indicate an interesting phenomenon that stimulants increasing only basolateral Cl^−^ conductance have no instantaneous influence on Cl^−^ secretion but induce a gradual decrease in Cl^−^ secretion (Figure 4A) caused by gradual diminution in ${\left[{Cl}^{-}\right]}_{i}$ (Figure 4B) due to an increase by an increase in a back flux of Cl^−^ to the basolateral space mediated through an increase in the basolateral Cl^−^ conductance.

#### A case where the membrane potential is changed (*V* ≠ *V*^0^) by application of compounds affecting the membrane conductance without any effects on Cl^−^ uptake via a pathway $\left(J_{C}\;=\;J_{C}^{0}:{CT}_{Act}\;=\;{CT}_{Act}^{0}\right)$ or apical or basolateral Cl^−^ conductance $(G_{A}\;=\;G_{A}^{0}$ and $G_{B}\;=\;G_{B}^{0})$

**Figure 5:**
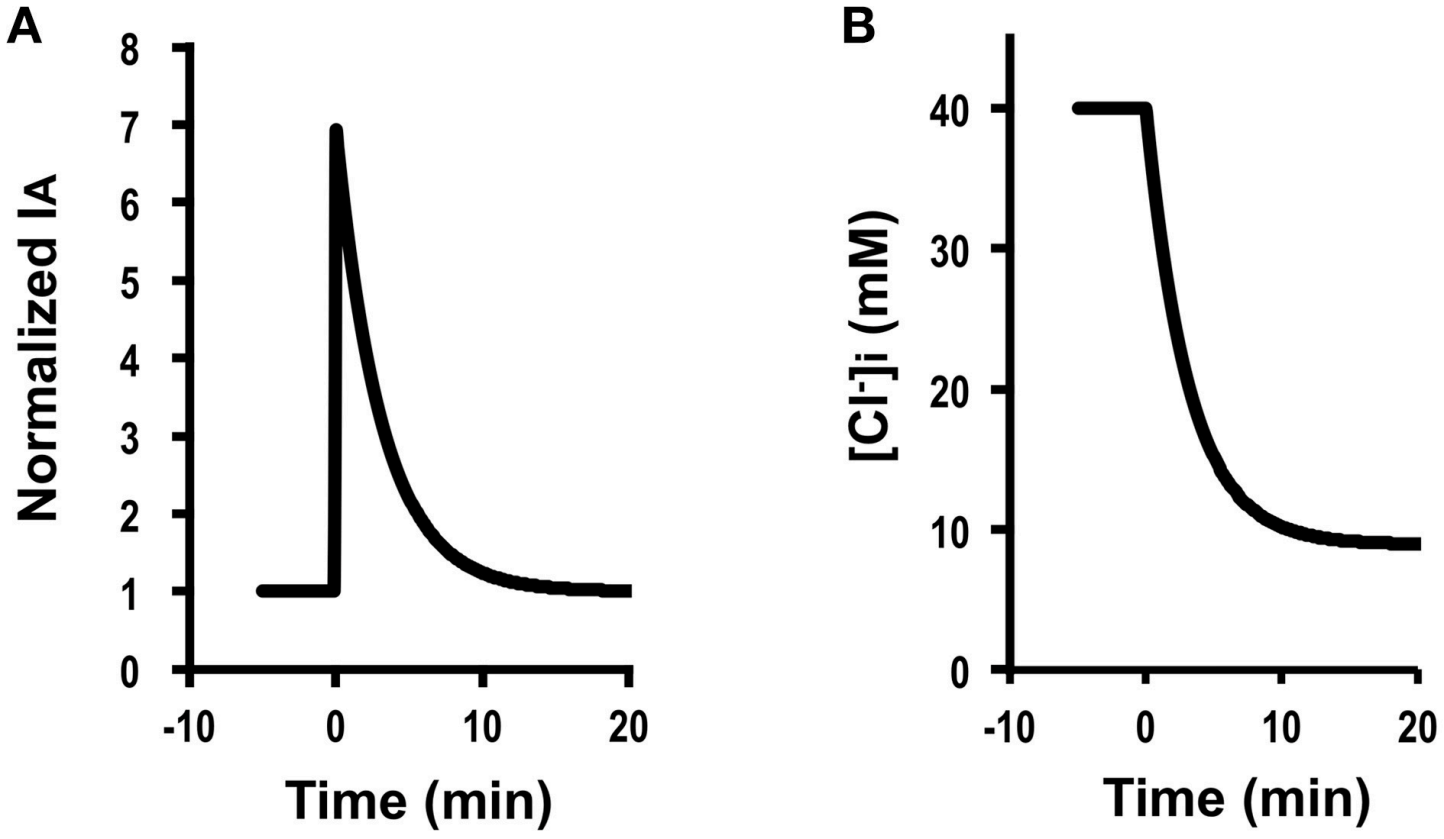
**Simulation of Cl^−^ secretion (*I*_*A*_) and ${\left[{Cl}^{-}\right]}_{i}$ under a condition where the membrane potential is changed (*V*≠*V*^0^; e.g., *V*^0^ = −40 mV and *V* = −70 mV) by application of compounds affecting the membrane potential without any effects on Cl^−^ uptake via a pathway ($J_{C}=J_{C}^{0}$) or the apical or basolateral Cl^−^ conductance ($G_{A}=G_{A}^{0}$ and $G_{B}=G_{B}^{0}$; $G_{B}^{0}=30\;G_{A}^{0}$)**. **(A)** A change in the membrane potential from −40 to −70 mV at time = 0 min causes a transient increase in Cl^−^ secretion (*I*_*A*_) about 6.8-fold [Equation (A18)] just after the change of the membrane potential. Then, Cl^−^ secretion (*I*_*A*_) gradually decreases, reaching a steady state; *I*_*A*_ (∞) is identical to an initial value of Cl^−^ secretion, $I_{A}^{0}$; $I_{A}\;\left(\infty \right)=I_{A}^{0}$ [see Equation (A18)]. **(B)** Simulation of [*Cl*${}^{-}{]}_{i}$. [*Cl*${}^{-}{]}_{i}$ in cells secreting Cl^−^ secretion (*I*_*A*_) shown in **(A)** decreases after a change in the membrane potential from −40 to −70 mV at time = 0 min.

For example, we calculated *I*_*A*_ (0) when V changes to −70 mV (*V* = −70 mV) from −40 mV (*V*^0^ = −40 mV) due to activation of K^+^ channel at the basolateral membrane at 25°C, the instantaneous Cl^−^ secretion (*I*_*A*_ (0)) is as follows (see Equation A18) using the information on $I_{A}^{0}$ (0.08 ± 0.02 μA/cm^2^, *n* = 9; Table 2). *I*_*A*_ (0) = −0.08 $\frac{-0.07}{-0.04}\;\frac{1-0.2104}{1-0.0654}\;+0.12\;\frac{-0.07\;\cdot \;325}{1-0.0654}\;\left(0.2104-0.0654\right)=-0.5418\;\left(\mu \text{A}∕{\text{cm}}^{2}\right)$. As mentioned in the section of Method, it is notable that the value of *I*_*A*_ has a negative one, however *I*_*A*_ shown in figures is converted to a positive one. This means that only a change in the membrane potential from −40 to −70 mV causes a transient increase in Cl^−^ efflux (*I*_*A*_(*t*)) about 6.8-fold [Equation (A18)] just after the change of the membrane potential followed by a decline toward the value before the change in the membrane potential ($I_{A}^{0}$) with exponential function with τ of $\frac{\text{CvF}\left(\text{exp}\left(\frac{\text{F}\text{V}}{\text{RT}}\right)-1\right)\;{\left[{\text{Cl}}^{-}\right]}_{o}}{V\left(G_{A}^{0}+G_{B}^{0}\right)}$ (Equation (A14) in Appendix; Figure 5A). This decline of *I*_*A*_(*t*) with τ of $\frac{1}{29}\frac{\text{CvF}\left(\text{exp}\left(\frac{\text{F}\text{V}}{\text{RT}}\right)-1\right)\;{\left[{\text{Cl}}^{-}\right]}_{o}}{{VG}_{A}^{0}}$ [Equation (A14) in Appendix] is due to a decrease in ${\left[{Cl}^{-}\right]}_{i}$ (Figure 5B) caused by hyperpolarization of the apical and basolateral.

**Table 2:** **Cl^−^ currents, and apical and basolateral conductance under basal and forskolin (FK)-stimulated conditions**.

Cl^−^ current under the basal condition ($I_{A}^{0}$)	0.08 ± 0.02 μA/cm^2^
Cl^−^ current under the FK-stimulated condition (*I*_*A*_(∞))	6.40 ± 0.14 μA/cm^2^
Apical conductance under the basal condition ($G_{A}^{0}$)	12.87 ± 1.31 μS
Basolateral conductance under the basal condition ($G_{B}^{0}$)	360.00 ± 57.42 μS
Apical conductance under the FK-stimulated condition ($G_{A}^{\infty }$)	689.04 ± 39.28 μS
Basolateral conductance under the FK-stimulated condition ($G_{B}^{\infty }$)	480.47 ± 40.27 μS

G_A_ and G_B_ shown here are respectively total conductance of apical and basolateral membrane of A6 cells cultured in Transwell-Clear permeable supports (0.33 cm^2^). Each character with superscripted 0 means its value under the basal condition at a steady state. Each character with superscripted ∞ means its value at a steady state after application of compounds affecting transcellular Cl^−^ secretion, n = 9.

#### A case where Cl^−^ uptake via a pathway (*J*_*C*_) changes $\left(J_{C}\neq J_{C}^{0}:\;{CT}_{Act}\neq {CT}_{Act}^{0}\right)$ time-dependently after application of compounds affecting intracellular environments modifying Cl^−^ secretion without any effects on apical or basolateral Cl^−^ conductance $(G_{A}=G_{A}^{0}$ and $G_{B}=G_{B}^{0})$ or membrane potential (*V* = *V*^0^)

**Figure 6:**
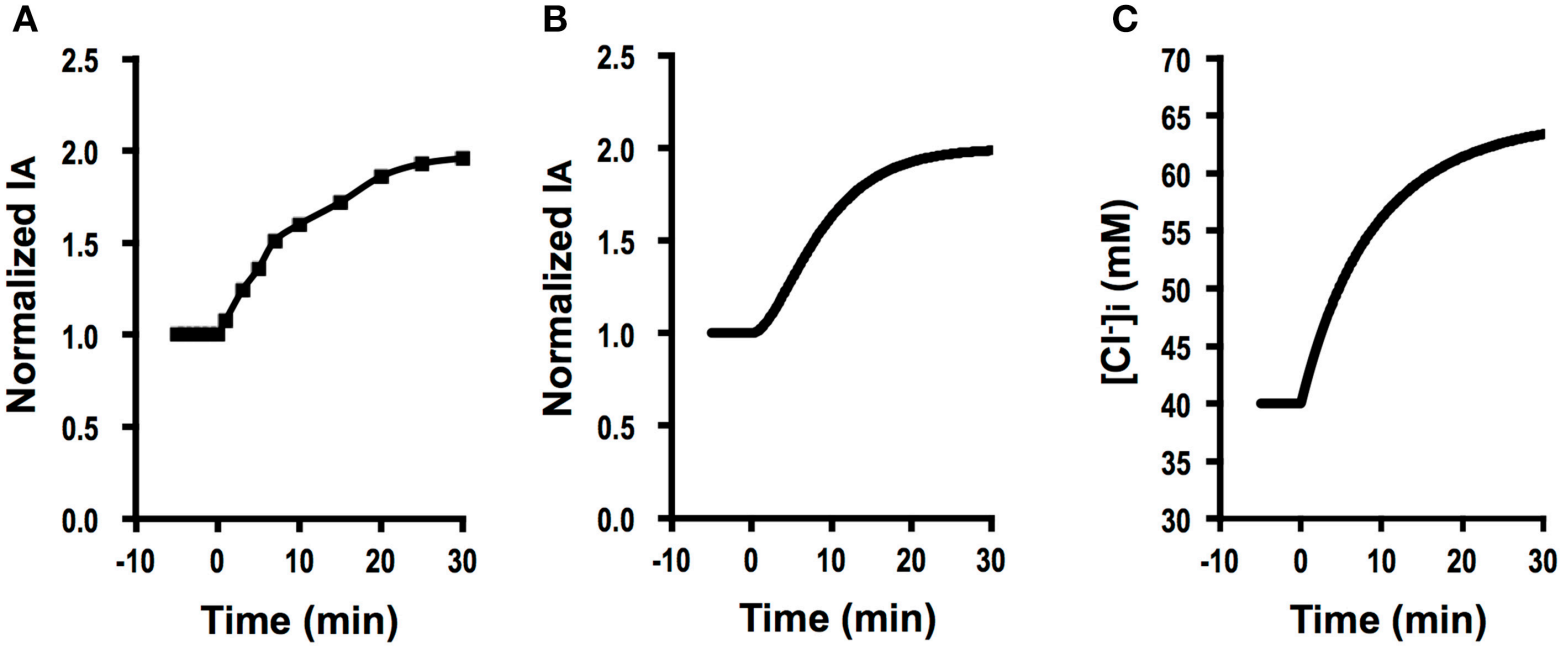
**The effect of apigenin on Cl^−^ secretion (*I*_*A*_) and simulation of Cl^−^ secretion (*I*_*A*_) and ${\left[{Cl}^{-}\right]}_{i}$ under a condition where Cl^−^ uptake via a pathway (*J*_*C*_) changes ($J_{C}\left(\infty \right)\;=\;2J_{C}^{0}:\;{CT}_{Act}\left(\infty \right)\;=\;2{CT}_{Act}^{0}$) after application of compounds affecting intracellular environments modifying Cl^−^ secretion without any effects on apical or basolateral Cl^−^ conductance ($G_{A}=G_{A}^{0}$ and $G_{B}=G_{B}^{0}$) or membrane potential (*V* = *V*^0^)**. **(A)** The effect of apigenin on Cl^−^ secretion (*I*_*A*_). Apigenin (100 μM) applied to the apical and basolateral solutions at time = 0 min induced a gradual increase in *I*_*A*_ reaching a steady level larger than the basal one. **(B)** Simulation of *I*_*A*_ in cells secreting Cl^−^ secretion (*I*_*A*_) shown in **(A)**. A stimulant is applied at time = 0 min. $I_{A}\;\left(0\right)=I_{A}^{0}$ [see Equation (A18)]. Then, Cl^−^ secretion (*I*_*A*_) gradually increases reaching a steady state; $I_{A}\left(\infty \right)=2\;I_{A}^{0}$ [see Equation (A18)]. **(C)** Simulation of ${\left[Cl^{-}\right]}_{i}$ in cells secreting Cl^−^ secretion (*I*_*A*_) shown in **(B)**. A stimulant is applied at time = 0 min. ${\left[Cl^{-}\right]}_{i}$ gradually increases after a two-fold change in *J*_*C*_ ($J_{C}\left(\infty \right)=2\;J_{C}^{0}:\;{CT}_{Act}\left(\infty \right)=2{CT}_{Act}^{0}$).

Figure 6A shows Cl^−^ secretion (*I*_*A*_) observed in A6 cells treated with 100 μM apigenin, which induced a gradual increase in *I*_*A*_, reaching a steady level larger than its initial level. Figure 6B shows the time course of Cl^−^ secretion under this condition simulated by using Equation (A18) (Appendix) in response to two-fold increases in Cl^−^ uptake via a pathway, *J*_*C*_, $\left(J_{C}\left(\infty \right)=\;2\;J_{C}^{0}:\;{CT}_{Act}\left(\infty \right)=2{CT}_{Act}^{0}\right)$ after application of a compound affecting intracellular environments modifying Cl^−^ secretion with $G_{A}=G_{A}^{0}$, $G_{B}=G_{B}^{0}$ and *V* = *V*^0^. $I_{A}\left(0\right)=I_{A}^{0}$ at $J_{C}\left(\infty \right)=\;2\;J_{C}^{0}$ (${CT}_{Act}\left(\infty \right)=2{CT}_{Act}^{0}$), $G_{A}=G_{A}^{0}$, $G_{B}=G_{B}^{0}$, and *V* = *V*^0^. Then, Cl^−^ secretion gradually increases reaching a steady state; $I_{A}\;\left(\infty \right)=2\;I_{A}^{0}$ [Equation (A18) in Appendix]. This simulation (Figure 6B) mimics Cl^−^ secretion in A6 cells treated with 100 μM apigenin (Figure 6A). Figure 6C shows a simulated change in ${\left[{Cl}^{-}\right]}_{i}$ of A6 cells showing Cl^−^ secretion shown in Figure 6B. The time course of ${\left[{Cl}^{-}\right]}_{i}$ increase is same as that of elevating phase of Cl^−^ secretion. Similar observations on Cl^−^ secretion in cells treated with quercetin or kaemferol (Cermak et al., 1998, 2002; Illek and Fischer, 1998; Asano et al., 2009; Zhang et al., 2011), suggesting that quercetin or kaemferol would induce Cl^−^ secretion gradually after their addition by activating NKCC1.

#### A case where Cl^−^ uptake via a pathway (*J*_*C*_) is time-dependently changed $\left(J_{C}\neq J_{C}^{0}:\;{CT}_{Act}\neq {CT}_{Act}^{0}\right)$ after application of compounds that affect intracellular environments modifying Cl^−^ secretion associated with changes in apical and basolateral Cl^−^ conductance $\left(G_{A}\neq G_{A}^{0}\right)$ and $\left(G_{B}\neq G_{B}^{0}\right)$ but without any change in the membrane potential (*V* = *V*^0^)

**Figure 7:**
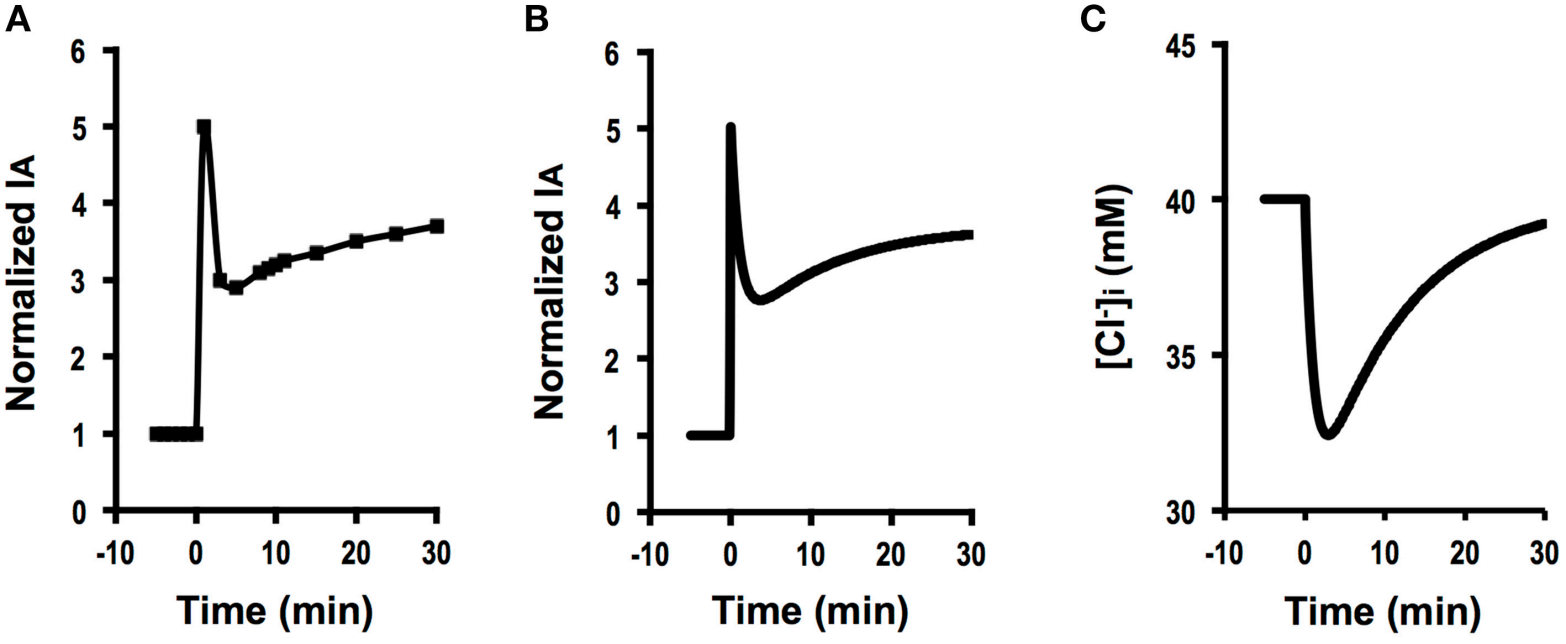
**The effect of genistein on Cl^−^ secretion (*I*_*A*_) and simulation of Cl^−^ secretion (*I*_*A*_) and ${\left[{Cl}^{-}\right]}_{i}$ under a condition where Cl^−^ uptake via a pathway *J*_*C*_ changes ($J_{C}\neq J_{C}^{0}$) time-dependently after application of compounds that affect intracellular environments modifying Cl^−^ secretion associated with changes in apical and basolateral Cl^−^ conductance ($G_{A}\neq G_{A}^{0}$ and $G_{B}\neq G_{B}^{0}$) but without any change in the membrane potential (*V* = *V*^0^)**. **(A)** The effect of genistein on Cl^−^ secretion (*I*_*A*_). Genistein (100 μM) applied to the apical and basolateral solutions at time = 0 min induced a biphasic increase in *I*_*A*_: (1) the first phase consists of an initial, transient increase in *I*_*A*_ followed by a decrease after application of a stimulant at time = 0, and 2) the second phase consists of a gradual increase in *I*_*A*_ after *I*_*A*_ reaches its minimum value at the first phase toward a steady level larger than the basal level. **(B)** Simulation of *I*_*A*_ in cells secreting Cl^−^ secretion (*I*_*A*_) shown in **(A)**. A stimulant is applied at time = 0 min. Cl^−^ secretion shows a five-fold increase just after application of a compound modifying Cl^−^ uptake via a pathway ($J_{C}=\;3\;J_{C}^{0}$), apical Cl^−^ conductance ($G_{A}=5\;G_{A}^{0}$) and basolateral Cl^−^ conductance ($G_{B}=4\;G_{B}^{0}$; $G_{B}^{0}=30\;G_{A}^{0}$) without any change in membrane potential (*V* = *V*^0^) according to Equation (A18). Then, Cl^−^ secretion gradually decreases from its peak. After reaching its minimum value, Cl^−^ secretion again increases toward a steady value shown in Equation (A18). **(C)** Simulated ${\left[Cl^{-}\right]}_{i}$ of cells secreting Cl^−^ shown in **(B)**. A stimulant is applied at time = 0 min. ${\left[Cl^{-}\right]}_{i}$ transiently decreases after increases in *G*_*A*_ and *G*_*B*_ followed by a gradual increase due to an increase in *J*_*C*_.

Genistein (100 μM) induced a biphasic increase in *I*_*A*_ in A6 cells (Figure 7A): (1) the first phase consisted of a transient increase in *I*_*A*_ followed by a decrease, and (2) at the second phase *I*_*A*_ gradually increased toward a steady level after reaching the minimum value at the first phase. We simulated this phenomenon of *I*_*A*_ observed in A6 cells treated with genistein (Figure 7A; Equation A18). There are two time constants ($\tau_{2}=\frac{1}{\alpha +\beta }\gg \tau_{1}=\frac{\text{CvF}\;\left(\text{exp}\left(\frac{\text{F}\text{V}}{\text{RT}}\right)-1\right)\;{\left[{\text{Cl}}^{-}\right]}_{\text{o}}}{V\left(G_{A}+G_{B}\right)}$) in the change in *I*_*A*_(*t*) as shown in Equations (A11) and (A14). Based on the inequality, $\tau_{2}=\frac{1}{\alpha +\beta }\gg \tau_{1}=\frac{\text{CvF}\;\left(\text{exp}\left(\frac{\text{F}\text{V}}{\text{RT}}\right)-1\right)\;{\left[{\text{Cl}}^{-}\right]}_{\text{o}}}{V\left(G_{A}+G_{B}\right)}$, the part of *I*_*A*_ contributing to the rising phase of *I*_*A*_(*t*) at the second phase is presented by Equation (A18) with a time constant of τ_2_, $\frac{1}{\alpha +\beta }$ [Equation (A11)].

Figure 7B shows the time course of Cl^−^ secretion in response to a compound causing $J_{C}\left(\infty \right)=\;3\;J_{C}^{0}\;\left({CT}_{Act}\left(\infty \right)=3{CT}_{Act}^{0}\right)$, $G_{A}=\;5\;G_{A}^{0}$ and $G_{B}=\;4\;G_{B}^{0}$ ($G_{B}^{0}=\;30\;G_{A}^{0}$) with no effect on membrane potential (*V* = *V*^0^) according to Equation (A18) in Appendix. $I_{A}\;\left(0\right)=5\;I_{A}^{0}$ (Equation A18 in Appendix). Then, *I*_*A*_ gradually decreases from its peak (Figure 7B). After reaching its minimum value, *I*_*A*_ again increases toward a steady value. This change in *I*_*A*_ shown in Figure 7B mimics Cl^−^ secretion presented in Figure 7A. Similar observations have been reported (Niisato et al., 1999; Deachapunya and Poonyachoti, 2013). Figure 7C shows a simulated change in ${\left[{Cl}^{-}\right]}_{i}$ of the cells secreting Cl^−^ presented in Figure 7B. This simulation indicates that ${\left[{Cl}^{-}\right]}_{i}$ decreases with a time course similar to that in the declining phase in Cl^−^ secretion following the initial, transient increase at the first phase due to a ${\left[{Cl}^{-}\right]}_{i}$ decrease caused by an increase in Cl^−^ secretion, and then increases due to elevation of Cl^−^ uptake via NKCC1, although ${\left[{Cl}^{-}\right]}_{i}$ shows no initial, transient increase unlike Cl^−^ secretion (*I*_*A*_; see Figures 7B,C). This simulation suggests us that genistein would have stimulatory action on both the apical Cl^−^ channel and the basolateral NKCC1.

**Figure 8:**
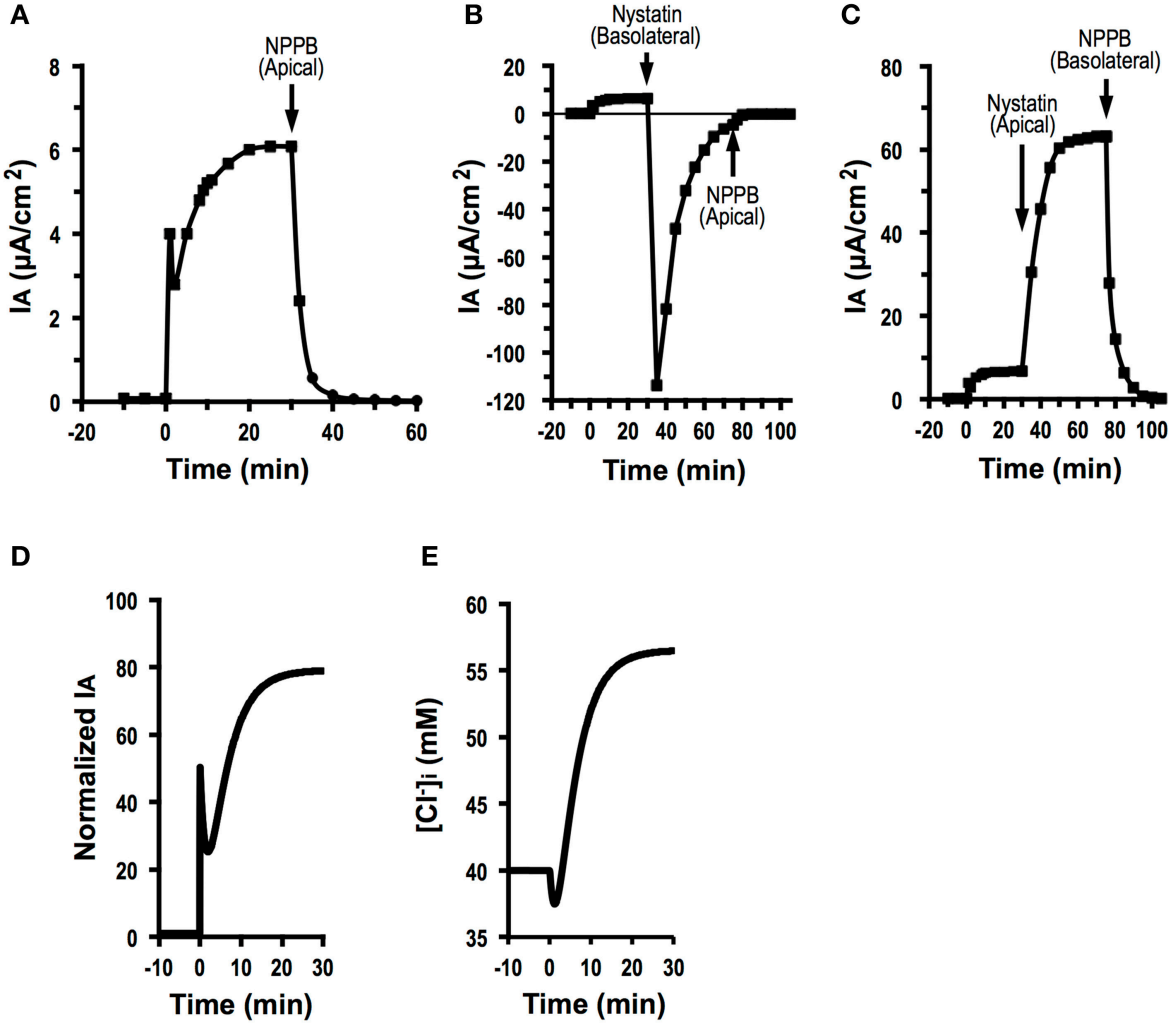
**Forskolin action on Cl^−^ secretion (*I*_*A*_), apical conductance (*G*_*A*_) and basolateral Cl^−^ conductance (*G*_*B*_) under an assumption with no change in membrane potential (*V* = *V*^0^), and simulation of Cl^−^ secretion (*I*_*A*_) and ${\left[Cl^{-}\right]}_{i}$**. **(A)** The effect of foskolin on Cl^−^ secretion (*I*_*A*_). Forskolin (10 μM) applied to the apical and basolateral solutions at time = 0 min induced an initial, transient increase in Cl^−^ secretion (*I*_*A*_ (0)) followed by a decrease. After reaching its minimum level, Cl^−^ secretion (*I*_*A*_) again increased toward a steady value. After Cl^−^ secretion (*I*_*A*_) reached a steady value, we applied 100 μM NPPB to the apical solution at time = 30 min, which diminished Cl^−^ secretion (*I*_*A*_). **(B)** Apical Cl^−^ conductance (*G*_*A*_) of A6 cells treated with forskolin. After application of forskolin (10 μM) at time = 0 min, we added nystatin (50 μM) to the basolateral solution to permeabilize the basolateral membrane at time = 30 min. Then, we applied 100 μM NPPB to the apical solution at time at 75 min. Under this condition, we determined the NPPB-sensitive conductance of apical membrane. **(C)** Basolateral Cl^−^ conductance (*G*_*B*_) of A6 cells treated with forskolin. After application of forskolin (10 μM) at time = 0 min, we added nystatin (50 μM) to the apical solution to permeabilize the apical membrane at time = 30 min. Then, we applied 100 μM NPPB to the basolateral solution at time 75 min. Under this condition, we determined the NPPB-sensitive conductance of basoalteral membrane. **(D)** Simulated Cl^−^ secretion (*I*_*A*_) of A6 cells treated with forskolin. A stimulant is applied at time = 0 min. We obtained $I_{A}\left(0\right)=50\;I_{A}^{0}$, $I_{A}\left(\infty \right)=79\;I_{A}^{0}$, $G_{A}=50\;G_{A}^{0}$ and $G_{B}\;=\;1.33\;G_{B}^{0}$ from this simulation [Equation (A18)]. **(E)** Simulated ${\left[Cl^{-}\right]}_{i}$ of cells secreting Cl^−^ shown in **(D)**. A stimulant is applied at time = 0 min. ${\left[Cl^{-}\right]}_{i}$ transiently decreases after application of forskolin followed by a gradual increase.

As shown in Equation (A19), we are able to determine $\frac{J_{C}\left(\infty \right)}{J_{C}^{0}}\left(=\frac{CT_{Act}\left(\infty \right)}{{CT}_{Act}^{0}}\;\right)$ by measuring $I_{A}^{o},\;I_{A}\left(\infty \right),\;G_{A}^{0},\;G_{B}^{0},\;G_{A}^{\infty }$, and $G_{B}^{\infty }$. We tried to determine the effect of fosrskolin (10 μM) on *J*_*C*_. After application of forskolin, *I*_*A*_ increased reaching an initial peak, then showed a decline phase followed by a gradual increase (Figure 8A). NPPB of 100 μM applied to the apical solution diminished *I*_*A*_ (Figure 8A). Statistical results indicate that $I_{A}^{o}$ was 0.08 ± 0.02 μA/cm^2^ (*n* = 9) and *I*_*A*_(∞) stimulated by forskolin (10 μM) was 6.40 ± 0.14 μA/cm^2^ (*n* = 9; Table 2). To measure the apical Cl^−^ conductance (*G*_*A*_), we permeabilized the basolateral membrane using nystatin. Forskolin (10 μM) applied at *t* = 0 increased *I*_*A*_. Nystatin (50 μM) added to the basolateral membrane at 30 min after application of forskolin induced *I*_*A*_ with a negative value (Figure 8B; see Discussion in detail). NPPB (100 μM) added to the apical solution at 45 min after application of nystatin (75 min after forskolin application in Figure 8B) diminished *I*_*A*_. Under this condition, we measured apical Cl^−^ conductance (*G*_*A*_) as the NPPB-sensitive conductance (Table 2). Similar to measurement of apical Cl^−^ conductance (*G*_*A*_), we also measured the basolateral Cl^−^ conductance (*G*_*B*_). As shown in Figure 8C, forskolin increased *I*_*A*_. Nystatin added to the apical membrane induced an increase in *I*_*A*_. NPPB (100 μM) added to the basolateral solution diminished *I*_*A*_. Under this condition, we measured basolateral Cl^−^ conductance (*G*_*B*_) as the NPPB-sensitive conductance (Table 2). The nystatin-induced increase in *I*_*A*_ was due to elevation of Na^+^, K^+^-pump current (see Discussion in detail). NPPB added to the basolateral solution decreased *I*_*A*_ by diminishing the Na^+^, K^+^-pump activity via blockade of the basolateral Cl^−^ channel (conductance; Niisato and Marunaka, 1999). Under this condition, we measured basolateral Cl^−^ conductance (*G*_*B*_) as the NPPB-sensitive conductance (Table 2). We also measured $G_{A}^{0}$ and $G_{B}^{0}$ without application of forskolin (Table 2) using a similar protocol mentioned above. Applying the values of $I_{A}^{o},\;I_{A}\left(\infty \right),\;G_{A}^{0},\;G_{B}^{0},\;G_{A}^{\infty }$, and $G_{B}^{\infty }$ shown in Table 2 to Equation (A19), we estimated the forskolin-induced activation of the electro-neutral ion transporter, NKCC1; the activity of NKCC1 was increased to 4.7-fold. Thus, we are able to estimate a relative change in an electro-neutral ion transporter, such as NKCC1, using the electrophysiological techniques combined with mathematical simulation proposed in the present study.

Figure 8D shows simulated Cl^−^ secretion (*I*_*A*_) of A6 cells treated with forskolin shown in Figure 8A. Figure 8E shows simulated [Cl^−^]_*i*_ of cells secreting Cl^−^ shown in Figure 8D. A transient increase in Cl^−^ secretion shown in Figure 8D is due to an increase in apical Cl^−^ conductance. A declining phase of Cl^−^ secretion following a transient increase in Cl^−^ secretion shown in Figure 8D is due to a decrease in [Cl^−^]_*i*_ (Figure 8E) caused by elevation of Cl^−^ secretion. A gradual increase in Cl^−^ secretion following the biphasic change in Cl^−^ secretion shown in Figure 8D is due to elevation of [Cl^−^]_*i*_ (Figure 8E) caused by activation of Cl^−^ transporter participating in uptake of Cl^−^ from the basolateral space.

## Discussion

In the present study, we report a mathematical model simulating transcellular Cl^−^ secretion combined with electrophysiological measurements. Using this method, we are able to provide information on regulation of the activity of Cl^−^ channels and transporters located at the apical and basolateral membranes contributing to the transcellular Cl^−^ secretion, and determine the activity of electro-neutral Cl^−^ transporter such as NKCC1.

We assumed that activity of Cl^−^ transporter, NKCC1, contributing to Cl^−^ uptake across the basolateral membrane does not depend on ${\left[{Cl}^{-}\right]}_{i}$, although activity of NKCC1 depends on the chemical potential difference between ${\left[{Cl}^{-}\right]}_{o}$ and ${\left[{Cl}^{-}\right]}_{i}$ in fact. In the present study, ${\left[{Cl}^{-}\right]}_{o}$ did not change. This means that we should consider modification of NKCC1 activity by ${\left[{Cl}^{-}\right]}_{i}$. However, the NKCC1-mediated ion transport is driven by electrochemical potential of Na^+^ between the intracellular and basolateral spaces across the basolateral membrane. This driving force of Na^+^ is much larger than chemical potential of Cl^−^, even that NKCC1 participates in two Cl^−^ uptake associated with one Na^+^ uptake. Therefore, this mathematical model proposed in the present study provides some valuable information, although the mathematical model proposed in the present study has limitation and we should consider a mathematical model including ${\left[{Cl}^{-}\right]}_{i}$-dependent regulation of NKCC1 activity like other studies dealing with this point (Weinstein and Krahn, 2010; Weinstein, 2010a,b).

We measured the apical Cl^−^ conductance (*G*_*A*_) by pemeabilizing the basolateral membrane applying nystatin to the basolateral solution (Figure 8B). Application of nystatin to the basolateral solution transiently induced *I*_*A*_ with a negative value (Figure 8B), which would mean Cl^−^ absorption (Cl^−^ influx across the apical membrane). This basolateral application of nystatin initially induces depolarization of the basolateral membrane by increasing conductance for monovalent ions including Na^+^, K^+^, and Cl^−^, associated with a gradual increase in ${\left[{Cl}^{-}\right]}_{i}$. Depolarization of basolateral membrane, in general, induces depolarization of apical membrane in some extent dependent on resistance (conductance) of tight junction (Marunaka, 2014a). This means that nystatin applied to the basolateral solution would initially induce Cl^−^ influx (absorption) from the apical solution via depolarization of apical membrane (see an initial, transient change in *I*_*A*_ with a negative value shown in Figure 8B). After nystatin initially induces depolarization of basolateral membrane by increasing conductance for monovalent ions including Na^+^, K^+^, and Cl^−^, ${\left[{Cl}^{-}\right]}_{i}$ would gradually increase via increment of Cl^−^ influx due to nystatin-induced depolarization of membrane associated with elevation of Cl^−^ conductance. This increase in ${\left[{Cl}^{-}\right]}_{i}$ abolishes Cl^−^ movement across the apical membranes (abolishment of Cl^−^ movement (*I*_*A*_) observed after the nystatin-induced transient change in *I*_*A*_ value shown in Figure 8B). On the contrary, apical application of nystatin had an opposite effect on *I*_*A*_: apical application of nystatin induced an increase in *I*_*A*_ with a positive value. As mentioned above, *I*_*A*_ increase caused by apical application of nystatin was due to elevation of the Na^+^, K^+^-pump current mediated by nystatin-induced increment of the intracellular Na^+^ concentration via an increase in Na^+^ influx across the apical membrane (Niisato and Marunaka, 1999).

Since NPPB of 100 μM used in the present might block other types of ion channels in addition to Cl^−^ channels (Kucherenko et al., 2013), we should consider a possibility that the Cl^−^ conductance measured as the NPPB-sensitive conductance would be overestimated and NPPB would affect epithelial ion transport by blocking some ion channels in addition to blockade of Cl^−^ channels. Our previous study using A6 cells (Niisato and Marunaka, 1999) has clearly indicated that the NPPB-sensitive conductance is compatible with the Cl^−^ conductance measured by replacement of Cl^−^ with an anion such as gluconate much less permeable to Cl^−^ channels. Therefore, at least in A6 cells the NPPB-sensitive conductance could be used as the Cl^−^ conductance.

A mathematical model of rat ascending Henle limb (Weinstein and Krahn, 2010; Weinstein, 2010a,b) simulates activity of NKCCs depending on concentrations of Na^+^, K^+^ and Cl^−^ in detail, and Cl^−^ flux mediated via NKCCs. However, no information is available on unidirectional epithelial Cl^−^ transport (Cl^−^ secretion) mediated via NKCCs and Cl^−^ channels expressed in polarized epithelial cells from these studies (Weinstein and Krahn, 2010; Weinstein, 2010a,b), although these studies provide activity characteristics of various types of ion transporters and channels dependent on concentrations of Na^+^, K^+^, and Cl^−^ in renal epithelial cells. Similar to our observation shown in Figure 2A, Hartmann and Verkman indicate that an increase in apical Cl^−^ conductance induces a biphasic change in Cl^−^ secretion: sudden elevation of apical Cl^−^ conductance causes an immediate increase in Cl^−^ secretion followed by a slower decrease to a level larger than the baseline at a steady state (Hartmann and Verkman, 1990). They report that the slow decrease in Cl^−^ secretion following the immediate increase caused by elevation of apical Cl^−^ conductance would be due to a decrease in [Cl^−^]_*i*_ mediated through the increase in Cl^−^ secretion based on elevation of apical Cl^−^ conductance (Hartmann and Verkman, 1990). Figure 2C clearly indicates a gradual decrease in [Cl^−^]_*i*_, which diminishes a chemical gradient for Cl^−^ secretion leading to a decrease in Cl^−^ secretion with a slower time course, strongly supporting the report by Hartmann and Verkman (1990). The model proposed by Hartmann and Verkman (1990) contains apical Cl^−^ conductance and basolateral NKCC, but not basolateral Cl^−^ conductance, while a model proposed in the present study contains basolateral Cl^−^ conductance contributing to a back flux of Cl^−^. Simulation of Cl^−^ secretion shown in Figure 2 proposes no change in Cl^−^ uptake, but a larger increase in apical Cl^−^ conductance than that in basolateral Cl^−^ conductance ($\frac{G_{A}^{\infty }}{G_{A}^{0}}>\frac{G_{B}^{\infty }}{G_{B}^{0}}$). This means that changes in apical and basolateral conductance under a condition of $\frac{G_{A}^{\infty }}{G_{A}^{0}}>\frac{G_{B}^{\infty }}{G_{B}^{0}}$ even without any change in Cl^−^ uptake increase Cl^−^ flux to the apical side associated with a decrease in Cl^−^ flux to the basolateral side by redistributing Cl^−^ fluxes to apical and basolateral sides dependent on a change in the ratio of $\frac{G_{A}^{\infty }}{G_{A}^{0}}$ and $\frac{G_{B}^{\infty }}{G_{B}^{0}}$. This phenomenon is able to explain the observation that Cl^−^ secretion at a steady state after stimulation is larger than the baseline Cl^−^ secretion reported by Hartmann and Verkman (1990). Fischer et al. (2007) have reported an observation in airway epithelial cells: application of CdCl_2_ or DIDS (Cl^−^ channel blockers) to the basolateral solution increases Cl^−^ secretion stimulated by forskolin. Further, a study (Duta et al., 2006) has indicated that a Cl^−^ channel blocker, DIDS, added to the basolateral solution elevates Cl^−^ secretion. He et al. (2011) has also reported that a Cl^−^ channel inhibitor, CaCCinh-A01, applied to the basolateral solution enhances Cl^−^ secretion in colonic epithelial tissues. Thus, these reports (Duta et al., 2006; Fischer et al., 2007; He et al., 2011) indicate that basolateral Cl^−^ conductance contributes to a back flux of Cl^−^: a decrease in basolateral Cl^−^ conductance with no change in apical Cl^−^ conductance ($\frac{G_{A}^{\infty }}{G_{A}^{0}}>\frac{G_{B}^{\infty }}{G_{B}^{0}}$) stimulates Cl^−^, supporting the model proposed in the present study. Further, Hartmann and Verkman indicate that a stimulant-induced Cl^−^ secretion in cells with smaller cell volume shows a faster change compared to that in cells with larger cell volume (Hartmann and Verkman, 1990). The present study also clearly showed that τ of change in *I*_*A*_ (*t*) is in direct proportion to cell volume [see Equation (A14)], supporting quantitatively the report by Hartmann and Verkman (1990).

## Conclusion

The present study provides an idea how transepithelial Cl^−^ secretion is modified by changes in activity of Cl^−^ channels and transporters and a method for determining changes in the activity of electro-neutral Cl^−^ transporters. The ideas and methods demonstrated in the present study provide powerful tools for the clarification of the regulatory mechanism of transepithelial Cl^−^ secretion, and very useful for development of new drugs modifying epithelial Cl^−^ secretion, although the mathematical model proposed in the present study has limitation to be adaptable to Cl^−^ secretion measured in living epithelial cells only under a short-circuit condition.

## Funding

This work was supported by Grants-in-Aid from Japan Society of the Promotion of Science (24590283 and 15K08183 to NN, 25893201 and 26713008 to AT, 25670111 and 15K15034 to YM), Salt Science (1235 to YM and NN, 1429 and 1542 to AT), KIT-KPUM-KPU-KPhU Collaborative Research Grant (2013 and 2015) to YM, NN and AT, Society for Research on Umami Taste, Nestlé Nutrition Council, Japan to AT, Kyoto Prefectural Public University Corporation to AT, Kyoto-Funding for Innovation in Health-related R&D Fields to YM and NN, Fuji Foundation for Protein Research to YM and NN, and Cell Research Conference to YM.

### Conflict of interest statement

The authors declare that the research was conducted in the absence of any commercial or financial relationships that could be construed as a potential conflict of interest.

## Appendix

We here show a model of transcellular Cl^−^ secretion in epithelial tissues (Figure 1). This model consists of three Cl^−^ moving pathways: (1) a releasing pathway of Cl^−^ from the intracellular space into the apical one across the apical membrane (**Pathway A)**; (2) a releasing pathway of Cl^−^ from the intracellular space into the basolateral one (**Pathway B**); (3) an uptake pathway of Cl^−^ from the basolateral space into the intracellular space, such as NKCC1, across the basolateral membrane (**Pathway C**). The transcellular Cl^−^ secretion is mediated by the following pathways: (1) Cl^−^ is taken up into the intracellular space via **Pathway C**; (2) Cl^−^ taken up into the intracellular space by **Pathway C** is respectively released into the apical and basolateral spaces via **Pathways A,B**; Cl^−^ taken up by **Pathway C** released into the apical space via **Pathway A** only contributes to the transcellular Cl^−^ secretion. The transcellular Cl^−^ secretion is generally measured as short-circuit currents (*I*_*SC*_) in Ussing chamber (Ussing and Zerahn, 1951). Short-circuit currents (*I*_*SC*_) are measured under a voltage clamp condition with the apical membrane potential identical to the basolateral membrane potential (*V*_*A*_ = *V*_*B*_) and all ionic compositions of the apical solution identical to those of basolateral solution. A change in ${\left[{Cl}^{-}\right]}_{i}$ with time (= *f*(*t*)) is presented as a differential equation, Equation (A1). Based on Goldman-Hodgkin-Katz equation (Goldman, 1943; Hodgkin and Katz, 1949), Cl^−^ fluxes across the apical and basolateral membranes are respectively shown as Equations (A2) and (A3). We applied these equations to the present study on an assumption that the extracellular and intracellular ions and fluids stirred completely, instantly just after their movements across the membrane. Explanation on the parameters used in simulation in the present study is shown in Table 1.

(A1) $$\begin{array}{r} \frac{df\left(t\right)}{dt}=-\frac{{\text{S}}_{\text{A}}J_{A}+{\text{S}}_{\text{B}}J_{B}+{\text{S}}_{\text{B}}J_{C}}{\text{Cv}}(1) \end{array}$$

(A2) $$\begin{array}{r} J_{A}=-\frac{P_{A}\text{F}V}{\text{RT}}\;\frac{f\left(t\right)-{\left[{\text{Cl}}^{-}\right]}_{\text{o}}\exp\left(\frac{\text{F}V}{\text{RT}}\right)\;}{1-\exp\left(\frac{\text{F}V}{\text{RT}}\right)\;} \end{array}$$

(A3) $$\begin{array}{r} J_{B}=-\frac{P_{B}\text{F}V}{\text{RT}}\;\frac{f\left(t\right)-{\left[{\text{Cl}}^{-}\right]}_{\text{o}}\exp\left(\frac{\text{F}V}{\text{RT}}\right)\;}{1-\exp\left(\frac{\text{F}V}{\text{RT}}\right)\;} \end{array}$$

where ${\left[{\text{Cl}}^{-}\right]}_{\text{o}}$ is the extracellular Cl^−^ concentration, S_A_ and S_B_ are respectively the areas of the apical and basolateral membranes, *J*_*A*_ and *J*_*B*_ are respectively the passive Cl^−^ movements across the unit area of the apical and basolateral membranes driven by electrochemical potential of Cl^−^ between the extracellular and intracellular spaces at time = *t*, *J*_*C*_ is the active Cl^−^ movement across the unit area of the basolateral membrane via Cl^−^ transporters such as NKCC1 driven by electrochemical potential of Na^+^ between the extracellular and intracellular spaces and is assumed to be independent of ${\left[{\text{Cl}}^{-}\right]}_{\text{i}}$ and the membrane potential, *P*_*A*_ and *P*_*B*_ are respectively permeability of Cl^−^ per unit areas of the apical and basolateral membranes, F is Faraday constant, *V* is the membrane potential (the apical membrane potential = the basolateral membrane potential under the short-circuit condition), R is the gas constant, and T is the absolute temperature. Here, *P*_*A*_S_A_ and *P*_*B*_S_B_ are respectively represented as Equations (A4) and (A5) on an assumption that *G*_*A*_ and *G*_*B*_ shown in the present study (specially measured under nystatin-permeabilized conditions) are subject to ${\left[{\text{Cl}}^{-}\right]}_{\text{o}}$ and independent of ${\left[{\text{Cl}}^{-}\right]}_{\text{i}}$ based on the relationship between permeability (*P*_*A*_ and *P*_*B*_) and conductance (*G*_*A*_ and *G*_*B*_) (Goldman, 1943) in order to simplify the derivation of Equation (A13):

(A4) $$\begin{array}{r} P_{A}{\text{S}}_{\text{A}}{\left[{\text{Cl}}^{-}\right]}_{\text{o}}=\frac{\text{RT}}{{\text{F}}^{2}}\;G_{A} \end{array}$$

(A5) $$\begin{array}{r} P_{B}{\text{S}}_{\text{B}}{\left[{\text{Cl}}^{-}\right]}_{\text{o}}=\frac{\text{RT}}{{\text{F}}^{2}}\;G_{B} \end{array}$$

where *G*_*A*_ and *G*_*B*_ are respectively the apical and basolateral Cl^−^ conductance. Combining Equations (A1)–(A5), we obtain Equation (A6).

(A6) $$\begin{array}{l} \frac{df(t)}{dt}=\frac{1}{\text{Cv}}[-\frac{V\left(G_{A}+G_{B}\right)}{\text{F}\left(\exp\left(\frac{\text{F}V}{\text{RT}}\right)-1\right)\;{\left[{\text{Cl}}^{-}\right]}_{\text{o}}}f(t)\\ \;+\;\frac{V\left(G_{A}+G_{B}\right)}{\text{F}\left(\exp\left(\frac{\text{F}V}{\text{RT}}\right)-1\right)}\exp\left(\frac{\text{F}V}{\text{RT}}\right)-{\text{S}}_{\text{B}}J_{C}(t)] \end{array}$$

where Cv is cell volume in m^3^: S_B_, area of basolateral membrane in m^2^; *V* is membrane potential in V (volt); *G*_*A*_ and *G*_*B*_ are respectively Cl^−^ conductance of apical and basolateral membrane in $\text{S }\left(=\frac{1}{\text{ V }}\frac{\text{Q}}{\text{ s }}\right)$: S is siemens, Q is coulomb and *s* is second. In the present study, we apply a condition that Cv, *V*, *G*_*A*_, and *G*_*B*_ after application of stimulants are constant without showing time-dependent changes to Equation (A6), even in the cases that stimulants affect *V*, *G*_*A*_, and *G*_*B*_: when a stimulant affects Cv, *V*, *G*_*A*_, and/or *G*_*B*_ the stimulant-induced changes in Cv *V*, *G*_*A*_, and/or *G*_*B*_ instantaneously reach steady levels keeping constant levels without showing time-dependent changes.

We further tried to establish a mathematical model regarding time-dependent change of *J*_*C*_ on assumptions that *J*_*C*_(*t*) is regulated by biochemical factors but not directly ${\left[{Cl}^{-}\right]}_{i}$, although activity of Cl^−^ transporters contributing to Cl^−^ uptake across the basolateral membrane (see Figure 1) depends on the chemical potential difference between ${\left[{\text{Cl}}^{-}\right]}_{\text{o}}$ and ${\left[{Cl}^{-}\right]}_{i}$ in fact. We considered a case that *S*_*B*_*J*_*C*_(*t*) (= j*CT*_*Act*_(*t*)) is determined by the following condition: (1) total amounts (*CT*_*T*_) of Cl^−^ transporters (*CT*) participating in Cl^−^ uptake across the basolateral membrane are unchanged, (2) the Cl^−^ transporter has active and inactive forms, (3) the inactive form (*CT*_*Inact*_) is converted to the active form with a rate of α^0^ under the basal condition or α after application of stimulant and the active form (*CT*_*Act*_) is converted to the inactive form with a rate of β^0^ under the basal condition or β after application of stimulant, (4) *CT*_*Act*_(*t*) and *CT*_*Inact*_(*t*) are respectively the amounts of the active and inactive forms of *CT* at time = *t* after application of stimulants and (5) j is the amount of Cl^−^ flux mediated by *CT*_*Act*_ per second (in mol/s). For example, α^0^ and α are respectively insertion rates of the Cl^−^ transporter into the basolateral membrane from the cytosolic store site under the basal condition and after application of stimulants, and β^0^ and β are respectively endocytotic rates of the Cl^−^ transporter from the basolateral membrane to the cytosolic space under the basal condition and after application of stimulants.

(A7) $$\begin{array}{r} CT_{Inact}\begin{array}{c} \alpha \\ ⇌\\ \beta \end{array}\;CT_{Act} \end{array}$$

(A8) $$\begin{array}{r} \frac{dCT_{Act}\left(t\right)}{dt}=\alpha \;CT_{Inact}\left(t\right)-\;\beta \;CT_{Act}\left(t\right) \end{array}$$

(A9) $$\begin{array}{r} {\text{CT}}_{\text{T}}=CT_{Inact}\left(t\right)+\;CT_{Act}\left(t\right) \end{array}$$

(A10) $$\begin{array}{l} S_{B}J_{C}(t)=\text{j }CT_{Act}(t)\\ \;=\text{j }[\frac{\alpha }{\alpha +\beta }{\text{CT}}_{\text{T}}-(\frac{\alpha }{\alpha +\beta }{\text{CT}}_{\text{T}}\\ \;-\;\frac{\alpha^{0}}{\alpha^{0}+\beta^{0}}{\text{CT}}_{\text{T}})\exp\left(-\frac{t}{\tau_{2}}\right)]\\ \;=\text{j }[CT_{Act}(\infty )-(CT_{Act}(\infty )\\ \;-CT_{Act}^{0})\exp\left(-\frac{t}{\tau_{2}}\right)] \end{array}$$

where ${CT}_{Act}^{0}=\frac{\alpha^{0}}{\alpha^{0}+\beta^{0}}{\text{CT}}_{\text{T}}$ is the amount of *CT*_*Act*_ at a steady state under the basal condition, ${CT}_{Act}\left(\infty \right)=\;\frac{\alpha }{\alpha +\beta }{\text{CT}}_{\text{T}}$ is the amount of *CT*_*Act*_ at a steady state after application of stimulants and τ_2_ is a time constant shown in Equation (A11) when *CT*_*Act*_(*t*) changes with time toward a steady state after application of stimulants.

(A11) $$\begin{array}{l} \tau_{2}=\frac{1}{\alpha +\beta }(11) \end{array}$$

When *J*_*C*_(*t*) (= *CT*_*Act*_(*t*)) is constant, α and β are respectively equal to α^0^ and β^0^ (α = α^0^ and β = β^0^). We obtain Equation (A12) by combining Equations (A6) and (A10).

(A12) $$\begin{array}{l} \frac{df(t)}{dt}=\frac{1}{\text{Cv}}[-\frac{V\left(G_{A}+G_{B}\right)}{\text{F}\left(\exp\left(\frac{\text{F}V}{\text{RT}}\right)-1\right){\left[{\text{Cl}}^{-}\right]}_{\text{o}}}f(t)\\ \;+\;\frac{V\left(G_{A}+G_{B}\right)}{\text{F}\left(\exp\left(\frac{\text{F}V}{\text{RT}}\right)-1\right)}\exp\left(\frac{\text{F}V}{\text{RT}}\right)-\text{j }CT_{Act}(\infty )\\ \;+\text{ j}\left(CT_{Act}(\infty )-CT_{Act}^{0}\right)\exp\left(-\frac{t}{\tau_{2}}\right)] \end{array}$$

Equation (A13) is obtained by resolving Equation (A12).

(A13) $$\begin{array}{l} f(t)=(f(0)-{\left[{\text{Cl}}^{-}\right]}_{\text{o}}\exp\left(\frac{\text{F}V}{\text{RT}}\right)\\ \;+\;\frac{\left(\text{exp}\left(\frac{\text{F}V}{\text{RT}}\right)-1\right){\left[{\text{Cl}}^{-}\right]}_{\text{o}}}{V\left(G_{A}+G_{B}\right)}\text{F j }CT_{Act}(\infty )\\ \;-\;\frac{\text{j }\left(CT_{Act}(\infty )-CT_{Act}^{0}\right)}{\text{Cv }\left(\frac{1}{\tau_{1}}-\frac{1}{\tau_{2}}\right)})\exp\left(-\frac{t}{\tau_{1}}\right)\\ \;+\text{​​}{\left[Cl^{-}\right]}_{\text{o}}\exp\left(\text{​}\frac{\text{F}V}{\text{RT}}\text{​}\right)-\frac{\left(\text{exp}\left(\frac{\text{F}V}{\text{RT}}\right)-1\right){\left[{\text{Cl}}^{-}\right]}_{\text{o}}}{V\left(G_{A}+G_{B}\right)}\text{F j }CT_{Act}\left(\infty \right)\\ \;+\text{​}\frac{\text{j }\left(CT_{Act}(\infty )-CT_{Act}^{0}\right)}{\text{Cv }\left(\frac{1}{\tau_{1}}-\frac{1}{\tau_{2}}\right)}\exp\left(-\frac{t}{\tau_{2}}\right) \end{array}$$

where τ_1_ is a time constant shown in Equation (A14).

(A14) $$\begin{array}{l} \tau_{1}=\frac{\text{CvF}\left(\exp\left(\frac{\text{F}V}{\text{RT}}\right)\;-1\right)\;{\left[{\text{Cl}}^{-}\right]}_{\text{o}}}{V\left(G_{A}+G_{B}\right)} \end{array}$$

We obtain Equation (A15) by combining *I*_*A*_(*t*) = Z_Cl_F S_A_ *J*_*A*_(*t*) and Equations (A2), (A4), and (A13).

(A15) $$\begin{array}{l} I_{A}(t)=\frac{VG_{A}}{\left(1-\exp\left(\frac{\text{F}V}{\text{RT}}\right)\right){\left[{\text{Cl}}^{-}\right]}_{\text{o}}}[(f(0)-{\left[{\text{Cl}}^{-}\right]}_{\text{o}}\exp\left(\frac{\text{F}V}{\text{RT}}\right)\\ \;+\;\frac{\left(\exp\left(\frac{\text{F}V}{\text{RT}}\right)-1\right){\left[{\text{Cl}}^{-}\right]}_{\text{o}}}{V\left(G_{A}+G_{B}\right)}\text{F j }CT_{Act}\left(\infty \right)\\ \;-\;\frac{\text{j }\left(CT_{Act}(\infty )-CT_{Act}^{0}\right)}{\text{Cv }\left(\frac{1}{\tau_{1}}-\frac{1}{\tau_{2}}\right)})\exp\left(-\frac{t}{\tau_{1}}\right)\\ \;-\frac{\left(\text{exp}\left(\frac{\text{F}V}{\text{RT}}\right)-1\right){\left[{\text{Cl}}^{-}\right]}_{\text{o}}}{V\left(G_{A}+G_{B}\right)}\text{F j }CT_{Act}(\infty )\\ \;+\;\frac{\text{j }\left(CT_{Act}(\infty )-CT_{Act}^{0}\right)}{\text{Cv }\left(\frac{1}{\tau_{1}}-\frac{1}{\tau_{2}}\right)}\exp\left(-\frac{t}{\tau_{2}}\right)] \end{array}$$

In the present study, we define *I*_*A*_(*t*) generated by Cl^−^ effluxes (Cl^−^ movements from the intracellular space to the extracellular space) as a positive value based on characteristics of Cl^−^ movements described in a model (Figure 1), although the *I*_*A*_(*t*) generated by Cl^−^ efflux was originally defined as a negative current. Equation (A1) is written as Equation (A16) because of $\frac{df\left(t\right)}{dt}=0$ under the basal condition.

(A16) $$\begin{array}{l} {\text{S}}_{\text{A}}J_{A}^{0}+{\text{S}}_{\text{B}}J_{B}^{0}+{\text{S}}_{\text{B}}J_{C}^{0}=0 \end{array}$$

By combining Equations (A2)–(A5), and (A16), we obtain Equation (A17) on an assumption that ${\left[{Cl}^{-}\right]}_{i}$ at time = 0 (*f*(0)) does not change instantaneously just after application of compound affecting transcellular Cl^−^ secretion in epithelial cells: *f*(0) = *f*^0^ (*f*^0^ is ${\left[{Cl}^{-}\right]}_{i}$ under the basal condition without application of any compounds affecting transcellular Cl^−^ secretion in epithelial cells).

(A17) $$\begin{array}{l} f\left(0\right)=\frac{\text{F j }{CT}_{Act}^{0}\left(1-\exp\left(\frac{FV^{0}}{RT}\right)\right){\left[{\text{Cl}}^{-}\right]}_{\text{o}}}{V^{0}\;\left(G_{A}^{0}+G_{B}^{0}\right)}+{\left[{\text{Cl}}^{-}\right]}_{\text{o}}\exp\left(\frac{\text{F}V^{0}}{\text{RT}}\right) \end{array}$$

Using a method reported in our previous study (Miyazaki et al., 2008), we measured ${\left[{Cl}^{-}\right]}_{i}\;\left(=f^{0}\right)$, which was about 40 mM. *V*^0^ was measured to be approximately –40 mV by electrophysiological methods reported in previous reports (Tohda et al., 1994; Nilius et al., 1995; Marunaka et al., 1999).

By combining $I_{A}^{0}=\frac{G_{A}^{0}}{G_{A}^{0}+G_{B}^{0}}\text{F j }{CT}_{Act}^{0}$ and Equations (A10), (A15), and (A17), we obtain Equation (A18).

(A18) $$\begin{array}{l} I_{A}(t)\text{​​}=I_{A}^{0}[\frac{G_{A}}{G_{A}^{0}}(\frac{CT_{Act}\left(\infty \right)}{CT_{Act}^{0}}\left(\frac{G_{A}^{0}+G_{B}^{0}}{G_{A}+G_{B}}\right)\\ +\;\left(\frac{V}{V^{0}}\left(\frac{1-\exp\left(\frac{\text{F}V^{0}}{\text{RT}}\right)}{1-\exp\left(\frac{\text{F}V}{\text{RT}}\right)}\right)-\frac{CT_{Act}(\infty )}{CT_{Act}^{0}}\left(\frac{G_{A}^{0}+G_{B}^{0}}{G_{A}+G_{B}}\right)\right)\\ \exp\left(-\frac{t}{\tau_{1}}\right))+\frac{1}{\text{F}}(\frac{G_{A}^{0}+G_{B}^{0}}{G_{A}^{0}}\left(\frac{VG_{A}\left(\frac{CT_{Act}(\infty )}{CT_{Act}^{0}}-1\right)}{\left(1-\exp\left(\frac{\text{FV}}{\text{RT}}\right)\right){\left[{\text{Cl}}^{-}\right]}_{\text{o}}}\right)\\ \left(\frac{\exp\left(-\frac{t}{\tau_{2}}\right)-\exp\left(-\frac{t}{\tau_{1}}\right)}{\text{Cv }\left(\frac{1}{\tau_{1}}-\frac{1}{\tau_{2}}\right)}\right))]\\ +\left[\frac{V\;G_{A}}{1-\exp\left(\frac{\text{F}V}{\text{RT}}\right)}\left(\exp\left(\frac{\text{F}V^{0}}{\text{RT}}\right)-\exp\left(\frac{\text{F}V}{\text{RT}}\right)\right)\right]\exp\left(-\frac{t}{\tau_{1}}\right) \end{array}$$

*I*_*A*_(*t*) (Equation A18) represents the amount of Cl^−^ secretion as a current.

A case where Cl^−^ uptake via a pathway (*J*_*C*_) is changed ($J_{C}\left(t\right)\neq J_{C}^{0}$) time-dependently after application of compounds that affect intracellular environments modifying Cl^−^ secretion associated with changes in apical and basolateral Cl^−^ conductance ($G_{A}\neq G_{A}^{0}$ and $G_{B}\neq G_{B}^{0}$) but without the membrane potential (*V* = *V*^0^). Equation (A19) is derived from Equation (A18) under a condition with $J_{C}\left(t\right)\;\neq \;J_{C}^{0}$, $G_{A}\neq \;G_{A}^{0}$, $G_{B}\neq \;G_{B}^{0}$, *V* = *V*^0^, and τ_1_ ≠ τ_2_.

(A19) $$\begin{array}{l} \frac{J_{C}\left(\infty \right)}{J_{C}^{0}}=\frac{CT_{Act}\left(\infty \right)}{CT_{Act}^{0}}=\frac{I_{A}\left(\infty \right)}{I_{A}^{0}}\frac{1+\frac{G_{B}^{\infty }}{G_{A}^{\infty }}}{1+\frac{G_{B}^{0}}{G_{A}^{0}}} \end{array}$$
